# Efficient Detection of Stigmatizing Language in Electronic Health Records via In-Context Learning: Comparative Analysis and Validation Study

**DOI:** 10.2196/68955

**Published:** 2025-08-18

**Authors:** Hongbo Chen, Myrtede Alfred, Eldan Cohen

**Affiliations:** 1 Department of Mechanical and Industrial Engineering University of Toronto Toronto, ON Canada

**Keywords:** in-context learning, large language model, prompting strategy, text classification, zero-shot, few-shot, machine learning, fairness, stigmatizing language, electronic health record, artificial intelligence

## Abstract

**Background:**

The presence of stigmatizing language within electronic health records (EHRs) poses significant risks to patient care by perpetuating biases. While numerous studies have explored the use of supervised machine learning models to detect stigmatizing language automatically, these models require large, annotated datasets, which may not always be readily available. In-context learning (ICL) has emerged as a data-efficient alternative, allowing large language models to adapt to tasks using only instructions and examples.

**Objective:**

We aimed to investigate the efficacy of ICL in detecting stigmatizing language within EHRs under data-scarce conditions.

**Methods:**

We analyzed 5043 sentences from the Medical Information Mart for Intensive Care–IV dataset, which contains EHRs from patients admitted to the emergency department at the Beth Israel Deaconess Medical Center. We compared ICL with zero-shot (textual entailment), few-shot (SetFit), and supervised fine-tuning approaches. The ICL approach used 4 prompting strategies: generic, chain of thought, clue and reasoning prompting, and a newly introduced stigma detection guided prompt. Model fairness was evaluated using the equal performance criterion, measuring true positive rate, false positive rate, and *F*_1_-score disparities across protected attributes, including sex, age, and race.

**Results:**

In the zero-shot setting, the best-performing ICL model, GEMMA-2, achieved a mean *F*_1_-score of 0.858 (95% CI 0.854-0.862), showing an 18.7% improvement over the best textual entailment model, DEBERTA-M (mean *F*_1_-score 0.723, 95% CI 0.718-0.728; *P*<.001). In the few-shot setting, the top ICL model, LLAMA-3, outperformed the leading SetFit models by 21.2%, 21.4%, and 12.3% with 4, 8, and 16 annotations per class, respectively (*P*<.001). Using 32 labeled instances, the best ICL model achieved a mean *F*_1_-score of 0.901 (95% CI 0.895-0.907), only 3.2% lower than the best supervised fine-tuning model, ROBERTA (mean *F*_1_-score 0.931, 95% CI 0.924-0.938), which was trained on 3543 labeled instances. Under the conditions tested, fairness evaluation revealed that supervised fine-tuning models exhibited greater bias compared with ICL models in the zero-shot, 4-shot, 8-shot, and 16-shot settings, as measured by true positive rate, false positive rate, and *F*_1_-score disparities.

**Conclusions:**

ICL offers a robust and flexible solution for detecting stigmatizing language in EHRs, offering a more data-efficient and equitable alternative to conventional machine learning methods. These findings suggest that ICL could enhance bias detection in clinical documentation while reducing the reliance on extensive labeled datasets.

## Introduction

### Background

Electronic health records (EHRs) are comprehensive digital systems that capture, store, and facilitate the retrieval and analysis of detailed longitudinal information about a patient’s health status [1]. As a central repository of patient information, EHRs are indispensable for facilitating efficient communication and collaboration among medical professionals [2,3]. Given the widespread adoption of EHR systems in health care settings globally and their vital role in streamlining health care communication [4,5], concerns have emerged regarding the prevalence of stigmatizing language within these records [6-9]. Specifically, a recent large-scale study [10] reported that stigmatizing language appears in a substantial proportion of clinical notes, with prevalence ranging from 42.8% to 59.1% in history and physical notes, consultation notes, and discharge summaries.

Stigmatizing language in health care refers to language that carries negative connotations, labels patients negatively, attributes blame, or renders judgment based on their social identities, medical conditions, or personal experiences [11]. Such language can be explicit, as in the use of derogatory terms such as “junkie” or “alcoholic” [8]. However, it also frequently manifests in more implicit forms, including subtle judgments embedded in tone, phrasing, or contextual framing [7]. Research in social psychology has demonstrated that implicit biases, which refer to unconscious attitudes or stereotypes, can be reflected through people’s language [12,13]. In health care, implicit biases may surface through the language used to describe patients. For instance, labeling a patient as “noncompliant” may seem clinically objective, but it can implicitly stigmatize their behavior by suggesting poor self-management, rooted in preconceived notions or stereotypes, without considering contextual factors such as mental capacity, available resources, or external circumstances. Implicit biases reflect broader systemic inequities, as institutional practices and cultural norms often perpetuate and normalize the use of stigmatizing language in clinical settings [14,15]. Stigmatizing language has been frequently observed in clinical specialties such as substance use disorders, mental health conditions, diabetes, and obesity, where it has shaped both clinical terminology and professional attitudes [8,16-18]. This systemic perpetuation of stigmatizing language reinforces negative stereotypes and creates barriers to equitable health care, undermining patient trust and treatment outcomes. By embedding biased language into clinical notes, these unconscious attitudes influence immediate clinical decisions and may perpetuate negative stereotypes over time [15,19], as subsequent clinicians rely on prior documentation [20]. These patterns were confirmed in a recent analysis of 754 clinical notes containing stigmatizing language [21], which found that stereotyping accounted for 41.2% of instances, followed by labeling patients as difficult (15.3%), disapproval (9.6%), unilateral decision-making (7.3%), and questioning patient credibility (5.3%). Such findings underscore the importance of mitigating bias to advance health care equity.

The presence of stigmatizing language can profoundly impact the quality of care. A study [18] found that clinicians exposed to the stigmatizing term “substance abuser,” as opposed to the more neutral phrase “person with substance use disorder,” were more likely to view the patient as personally culpable and deserving of punitive action rather than treatment. In addition, a national survey of 655 emergency physicians, those who used the stigmatizing label “sickler” to describe patients with sickle cell disease, demonstrated lower compliance with national treatment guidelines and were less likely to prescribe appropriate medications [17]. Complementing these findings, previous research has also shown that physicians who read notes containing stigmatizing language are more likely to develop negative attitudes toward the patient and to provide less aggressive pain management [22]. Notably, only 18% of inpatient progress notes are manually entered, with the majority being imported or copied from previous documentation [20]. The reliance on previous documentation raises concerns about the potential perpetuation of stigmatizing language, which could reinforce negative biases and influence clinical decision-making over time. With the implementation of the 21st Century Cures Act in 2021 in the United States, patients have gained access to their EHRs [23], making these records increasingly integral to the clinician-patient relationship. A large survey study [24] found that 10% of patients feel judged or offended due to labeling and evidence of disrespect in EHRs. Another study [16] reported that 19% of patients indicated they would avoid future medical appointments if their physicians used stigmatizing language toward them, while 21% stated they would seek a new physician. There is also substantial evidence that stigmatizing language in EHRs can jeopardize the therapeutic relationship, leading to distrust, delayed care, and reduced treatment engagement [7,25,26].

Given the detrimental effects of stigmatizing language in EHRs and the substantial resources required for its manual identification, there is a growing interest in developing automated detection methods. Manual detection typically necessitates annotators possessing extensive clinical expertise to accurately interpret context, as well as linguistic proficiency to discern subtle indicators of stigma [15,27]. In current practice, manual annotation typically involves experienced physicians reading and classifying individual sentences or notes from EHRs, an approach that is labor-intensive and costly, particularly when scaled across large datasets [15,27,28]. To overcome these challenges, researchers have increasingly explored machine learning approaches for the automated detection of stigmatizing language within EHRs. For instance, Sun et al [15] developed a logistic regression classifier to assess whether sentences in EHRs convey stigmatization, using a proprietary dataset, while Harrigian et al [27] conducted a comparative analysis of logistic regression and Bidirectional Encoder Representations from Transformers (BERT) classifiers for the detection of stigmatizing language. However, these studies have all used supervised learning approaches, which generally require thousands to tens of thousands of annotated data points to achieve optimal performance. The assumption that such large volumes of annotated data are readily available is often unrealistic, as the acquisition of labels for stigmatizing language detection presents significant challenges. Stigmatizing language is frequently subtle, covert, and highly contextual. Consequently, acquiring annotated data for stigmatizing language detection can be demanding and resource intensive. The previous studies’ heavy reliance on supervised machine learning approaches has highlighted a significant limitation in addressing scenarios where annotated data may be scarce.

In-context learning (ICL) is an emerging paradigm for addressing natural language processing (NLP) tasks in data-scarce settings [29-32]. ICL enables general-purpose large language models (LLMs) to adapt to new tasks by incorporating instructions and either zero or a few examples directly within the input prompt, thereby reducing reliance on extensively annotated datasets [29,33,34]. By leveraging the robust contextual understanding capabilities inherent in general-purpose LLMs, the ICL approach is particularly well-suited for detecting stigmatizing language, a task that requires the identification of subtle nuances and a high level of contextual comprehension. To the best of our knowledge, no study has systematically examined the efficacy of the ICL approach for stigmatizing language detection, underscoring a significant gap in the current research landscape.

**Figure 1:**
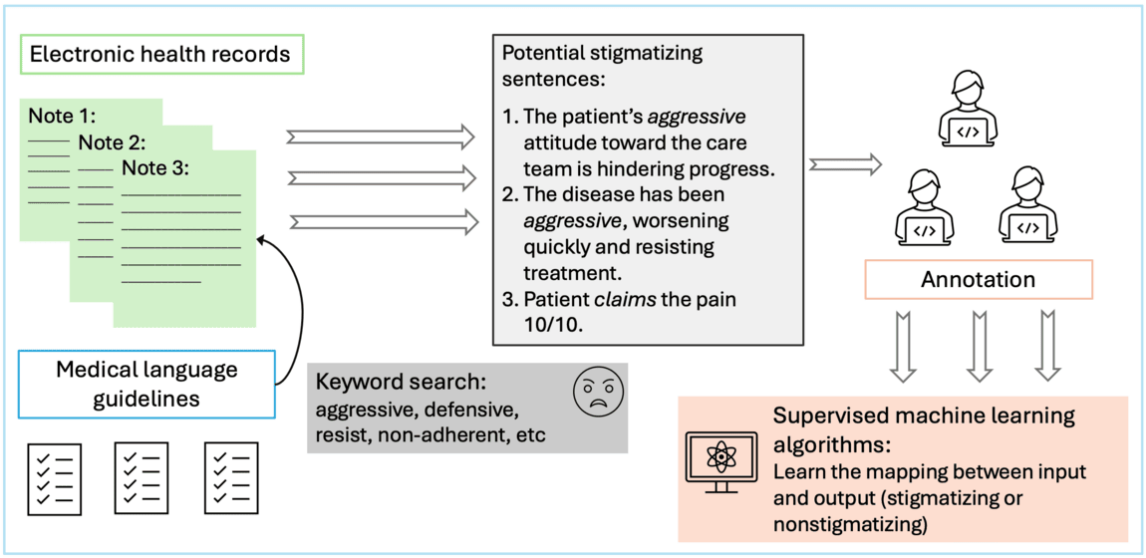
Workflow for developing a supervised machine learning model in stigmatizing language detection.

The effectiveness of the ICL approach is contingent upon prompt engineering, which involves crafting precise, informative, and contextually relevant prompts to guide the LLM in executing specific tasks [29,35,36]. Therefore, the efficacy of the ICL approach could be enhanced by a tailored prompting strategy for stigmatizing language detection. Previous studies on detecting stigmatizing language typically involve a 2-stage process (Figure 1): first, the extraction of sentences containing potentially stigmatizing words or phrases in accordance with medical language guidelines, followed by the development of the supervised machine learning model with the annotated data set [15,27]. The potentially stigmatizing words or phrases identified during the extraction process can provide valuable information that can be incorporated into the prompting strategy. By explicitly prompting the LLM to focus on these terms, we anticipate an improvement in its sensitivity to detecting stigmatizing language. Furthermore, given that stigmatizing language is often covert and subtle [27,37], incorporating common linguistic characteristics associated with such language may further increase the model’s accuracy in identifying instances of stigmatization. Customizing prompts to address the unique aspects of stigmatizing language presents a valuable opportunity to enhance detection precision.

While developing effective machine learning models to detect stigmatizing language is important, it is equally critical to ensure that these models do not introduce or perpetuate existing biases [38]. Numerous studies have demonstrated that machine learning classifiers can exhibit algorithmic bias, resulting in performance disparities across groups of patients with different protected attributes, such as sex, race, age, and socioeconomic status [39,40]. For example, Seyyed-Kalantari et al [41] found that a leading machine learning classifier for detecting chest X-ray pathologies exhibited a higher false negative rate for Hispanic female patients. In the context of stigmatizing language detection, algorithmic bias can lead to uneven identification and correction of stigmatizing language, disproportionately affecting certain groups and potentially exacerbating health disparities. To the best of our knowledge, no previous studies have specifically examined the fairness of classifiers in detecting stigmatizing language within EHRs, highlighting the need for further investigation into how these models perform across diverse patient populations. By rigorously evaluating the fairness of these models, we can identify potential disparities and ensure that they equitably benefit patients from diverse backgrounds.

### Study Objectives

We aimed to investigate the effectiveness of the ICL approach for detecting stigmatizing language in EHRs under data-scarce conditions. First, we evaluated the performance of the ICL approach against established approaches in zero-shot (no annotated data) and few-shot settings (a small amount of annotated data), including the textual entailment [42] and SetFit [43]. For the ICL approach, we used 4 distinct prompting strategies, including more sophisticated strategies, such as the chain of thought (COT) [44] and the clue and reasoning prompting (CARP) [30]. We also introduced a novel prompting strategy, termed “stigma detection guided prompt,” specifically designed to enhance the precision and effectiveness of detecting stigmatizing language. Second, to further contextualize ICL’s performance, we conducted a comparative analysis of supervised fine-tuning and ICL to evaluate the extent to which ICL can achieve competitive performance under data-scarce conditions relative to a well-established method that leverages a large corpus of annotated data. This comparison provides valuable insights for hospitals, enabling them to assess the cost-benefit trade-offs associated with investing in annotated datasets for detecting stigmatizing language in clinical documentation. Third, we assessed the fairness dimension of the classifiers used for detecting stigmatizing language in this study by comparing their performance across diverse patient groups characterized by various protected attributes, including sex, age, and race.

## Methods

### Dataset Description

In this study, we used an open-source stigmatization detection dataset prepared by Harrigian et al [27]. The dataset consists of 5043 sentences, of which 3249 (64.4%) are labeled as stigmatizing and 1794 (35.6%) as nonstigmatizing. The original creators randomly sampled these sentences from 4710 discharge summaries associated with 4259 patients within the Medical Information Mart for Intensive Care (MIMIC)–IV database and subsequently annotated them. The MIMIC-IV is an EHR dataset that comprises 331,794 discharge summaries from the Beth Israel Deaconess Medical Center in Boston, Massachusetts, United States, from 2008 to 2019 [45]. The observed prevalence of stigmatizing language in this dataset is notably higher than previously reported estimates, such as the 42.8% identified by Weiner et al [10]. This difference is attributable to the broader scope of stigmatizing expressions captured in the dataset prepared by Harrigian et al [27], which encompasses a wider range of terminology than earlier studies.

Discharge summaries were selected because they provide detailed documentation of the patient’s clinical course, including diagnoses, treatments, and physician observations, which are particularly relevant for investigating stigmatization in clinical notes. Sentences were extracted based on the presence of potentially stigmatizing words or expressions, such as “noncompliant” and “agitated.” A symmetric context window of 15 tokens on either side of each sentence was applied to preserve surrounding context and enhance model interpretation. If fewer than 15 tokens were available on either side, the context window was shortened as needed to fit the available text. Each sentence was independently annotated by 2 members of an annotation team comprising 1 clinician and 2 research assistants based on a predefined framework [27]. Any disagreements were resolved through discussion among all 3 annotators. The annotation process achieved a high level of interrater agreement, with an average pairwise Cohen κ exceeding 0.9. The annotation of stigmatization was determined based on framing and intent of the sentence with its surrounding context (ie, whether the sentence implies that the patient’s behavior is cast in a negative light). Sentences that did not describe patient behavior, or that neutrally described patient behavior, acknowledged structural or clinical barriers, or conveyed empathy from health care providers were not considered stigmatizing. The demographic information of the dataset, including patient sex, age, and race, is presented in Table 1. The “other or unknown” race category included individuals who did not report their race. A more detailed description of the dataset is provided in Multimedia Appendix 1 [15,18,19,22,27,37].

**Table 1:** Demographic information of the stigmatizing dataset used in this study.

Demographic attributes		Stigmatizing sentences, n (%)	Nonstigmatizing sentences, n (%)
**Sex**			
	Male (n=2600)	1731 (66.6)	869 (33.4)
	Female (n=2443)	1518 (62.1)	925 (37.9)
**Age (y)**			
	0-25 (n=468)	303 (64.7)	165 (35.3)
	26-50 (n=1639)	1184 (72.2)	455 (27.8)
	51-75 (n=2005)	1249 (62.3)	756 (37.7)
	>75 (n=931)	513 (55.1)	418 (44.9)
**Race**			
	Asian (n=125)	69 (55.2)	56 (44.8)
	Black (n=945)	676 (71.5)	269 (28.5)
	Hispanic (n=246)	159 (64.6)	87 (35.4)
	Other or unknown (n=522)	305 (58.4)	217 (41.6)
	White (n=3205)	2040 (63.7)	1165 (36.3)

### Ethical Considerations

This study used a publicly available dataset consisting of EHR sentences. All patient records were anonymized by the dataset’s original creators, ensuring that no individual identifiable information was accessible to the research team [45]. As such, the study is exempt from research ethics board review in accordance with Article 2.2 of the Tri-Council Policy Statement [46]. The original collection of data was approved by the institutional review boards of the Beth Israel Deaconess Medical Center, Boston, Massachusetts, and the Massachusetts Institute of Technology, Cambridge, Massachusetts (2001P001699). The institutional review boards also granted a waiver of informed consent and approved the sharing of the research resource, thereby permitting secondary analysis of the deidentified data without additional consent.

No compensation was provided or required for the use of this secondary data. No identification of individual participants or users in any images of the manuscript or supplementary material is possible. To access this dataset, the lead author fulfilled all necessary regulatory requirements, including the completion of the course “CITI data or specimens only research training” (certification number 62353094). In addition, the lead author signed a data use agreement to ensure the appropriate use of the data in compliance with relevant policies and regulations.

### Comparative Analysis for Stigmatizing Language Detection

Zero-shot and few-shot learning are machine learning paradigms that allow models to perform tasks with either no labeled data or with only a minimal set of labeled examples, respectively [47,48]. Zero-shot learning enables a model to perform a task without any previous examples, relying solely on its pretrained knowledge. In contrast, few-shot learning operates by providing the model with a limited number of labeled examples, typically ranging from 10 to 100.

In this study, we conducted a comprehensive evaluation of the effectiveness of the ICL approach for detecting stigmatizing language in EHRs under conditions of limited data availability. We compared the performance of ICL against the textual entailment approach [42] within a zero-shot learning context and the SetFit approach [43] within a few-shot learning context, specifically analyzing scenarios involving 4, 8, and 16 annotated examples per class. For the few-shot learning experiments, data points were randomly selected as examples. To ensure reproducibility and mitigate potential variability in performance due to random sampling, the selection process was repeated 5 times using distinct random seeds. The final performance results reported in this study represent the average across these 5 runs, providing a more robust and stable assessment of model performance.

In addition, we extended our comparative analysis to include a fully supervised fine-tuning approach. This broader comparison allows us to rigorously assess the strengths and potential limitations of the ICL approach in the detection of stigmatizing language within EHRs. All approaches used in this study adhered to the framework established in previous studies (Figure 1), wherein sentences were extracted based on potentially stigmatizing words or phrases identified from medical language guidelines, followed by the development of detection approaches (ie, ICL).

### Textual Entailment Approach

The textual entailment approach conceptualizes zero-shot text classification as an entailment task [42]. This method is among the most widely adopted for zero-shot text classification and has demonstrated state-of-the-art performance across various benchmarks [49,50]. In this approach, the input text is interpreted as a premise, and the model assesses whether this premise logically entails the corresponding class label. Specifically, in the context of stigmatizing language detection, the model first considers a sentence from an EHR as the premise and then evaluates whether it entails stigmatization or nonstigmatization. The textual entailment approach relies on the knowledge embedded in pretrained language models, which have been trained on natural language inference datasets [51]. This training effectively enhances the model’s capability for sentence comprehension. In our study, we used a selection of widely recognized pretrained language models for natural language inference to assess the effectiveness of the textual entailment approach in detecting stigmatizing language (Table 2). These models were selected due to their widespread use in zero-shot classification tasks [50,52,53] and their representation of a diverse range of transformer architectures, enabling us to fully explore the potential of the textual entailment approach for detecting stigmatizing language.

**Table 2:** Pretrained language models used in the textual entailment approach.

Abbreviation	Full model name
ROBERTA-M	roberta-large-mnli
BART-M	bart-large-mnli
DEBERTA-M	deberta-large-mnli
BERT-M	bert-base-multilingual-cased-multinli_tr

### SetFit Approach

SetFit is a popular few-shot text classification approach that has been applied across various domains [50,54,55]. The SetFit approach consists of a two-step process [43]. First, a pretrained sentence transformer is fine-tuned in a contrastive Siamese manner using pairs of labeled data. The objective of this contrastive fine-tuning is to ensure that embeddings of semantically similar sentences are close together in the feature space while embeddings of semantically dissimilar sentence pairs are maximally distanced. This process aims to generate meaningful representations that accurately capture the semantic relationships between sentences. In the second step, a classification head is trained using the representations produced by the fine-tuned sentence transformer. For this study, we used 6 pretrained sentence transformers to evaluate the effectiveness of the SetFit approach in detecting stigmatizing language (Table 3). MPNET-V2 and MINILM-V2 were the sentence transformers used in the original work that proposed SetFit [43], while the more contemporary E5-V2 and ROBERTA-V2 were included due to their superior performance on various NLP benchmarks [56,57]. We also incorporated 2 sentence transformers pretrained on medical domain knowledge: BERT-BIO-ST and BIOLORD. The inclusion of these models was based on the hypothesis that domain-specific pretraining may enhance the model’s ability to capture contextually relevant medical terminology and nuances, thereby improving its effectiveness in detecting stigmatizing language [58].

**Table 3:** Pretrained sentence transformers used in the SetFit approach.

Abbreviation	Full model name
MPNET-V2	paraphrase-mpnet-base-v2
E5-V2	e5-base-v2
ROBERTA-V2	stsb-roberta-base-v2
MINILM-V2	paraphrase-multilingual-MiniLM-L12-v2
BERT-BIO-ST	S-BioBert-snli-multinli-stsb
BIOLORD	BioLORD-2023

### Fully Supervised Fine-Tuning Approach

The fully supervised fine-tuning approach involves adapting a pretrained language model to a specific task by fine-tuning it on a labeled dataset, where each instance is associated with its corresponding label [29]. In this study, we used 5 pretrained language models to examine the efficacy of a fully supervised fine-tuning approach for detecting stigmatizing language (Table 4). ROBERTA, ROBERTA-XLM, and BERT were selected due to their extensive adoption and strong performance across text classification tasks in different domains [50,59-61]. BERT-BIO and BERT-biomed were included for their pretraining on biomedical text corpora, making them particularly well-suited for NLP tasks involving medical terminology [58,62]. We excluded certain contemporary medical pretrained language models, such as Gatortron [45] and ClinicalBERT [46], from our analysis. These models were omitted because they were pretrained on the MIMIC dataset, which could introduce data leakage and potentially lead to an overestimation of the model’s effectiveness. We specifically focused on fine-tuning encoder-based language models, such as BERT, rather than decoder-based models (ie, LLAMA) for 2 primary reasons. First, previous research has demonstrated that encoder-only models typically achieve superior or comparable performance to decoder-based models in text classification tasks with relatively small, labeled datasets, which aligns with our study setting [63,64]. Second, BERT-based models currently represent the state-of-the-art for supervised stigmatizing language detection [27]. Thus, using these models as a benchmark allows for a more rigorous evaluation of ICL performance against established methodologies.

**Table 4:** Pretrained language models used in the fully supervised fine-tuning approach.

Abbreviation	Full model name
ROBERTA	roberta-base
BERT	bert-base-uncased
BERT-BIO	biobert-v1.1
ROBERTA-XLM	xlm-roberta-base
BERT-BIOMED	BiomedNLP-BiomedBERT-base-uncased-abstract-fulltext

We fine-tuned these pretrained language models on 3543 labeled sentences from EHRs, representing approximately 70% of the total data. The supervised fine-tuning approach was included to provide a robust benchmark for comparison with the ICL approach in detecting stigmatizing language. Hyperparameters were tuned using a validation dataset of 500 data points, representing approximately 10% of the total dataset. Hyperparameter tuning was conducted for key parameters using grid search, including learning rate, batch size, and weight decay rate, to optimize model performance and mitigate overfitting. We used the AdamW optimizer [65] with default betas set to 0.9 and 0.999 and an epsilon of 1 × 10^-6^ to ensure numerical stability. The number of epochs was determined using an early stopping criterion, where training was terminated if the validation loss did not decrease for 5 consecutive epochs. The final hyperparameter settings were selected based on the model’s best performance on the validation dataset before being evaluated on the held-out test dataset.

### ICL Approach

The ICL approach leverages general-purpose LLMs through prompting techniques to rapidly adapt to a wide range of NLP tasks [29]. The approach has demonstrated exceptional performance in applications such as question answering, machine translation, text summarization, and text classification [29,32-34]. In this study, we used 5 widely used open-source general-purpose LLMs to evaluate the efficacy of the ICL approach in detecting stigmatizing language (Table 5). We refer to models such as LLAMA-3 as general-purpose LLMs to emphasize their broad applicability across various NLP tasks without extensive task-specific fine-tuning. We specifically selected instruction fine-tuned models, as previous research has shown that instruction fine-tuning significantly enhances model performance across a range of NLP tasks, particularly in zero-shot and few-shot settings [66,67]. In addition, we included BIO-LLAMA-3, a domain-specific model that has undergone instruction fine-tuning on a large biomedical dataset [68], to assess the potential benefits of domain adaptation in stigmatizing language detection. Due to the constraints of the MIMIC-IV data use agreement, we were unable to use newer cloud-based models, such as GPT-4 or Claude 3.5, as their use would necessitate sharing data with external parties. Previous studies have demonstrated that general-purpose LLMs are highly sensitive to subtle variations in prompt formatting [69,70]. To optimize the performance of the ICL approach, we devised a validation data set for prompt format tuning, which included adjustments such as the wording and positioning of instructions. A random sample of 30 data points was selected to construct this validation set, designed to replicate a data-scarce scenario consistent with previous research [71,72].

**Table 5:** General-purpose large language models (LLMs) used in the in-context learning approach.

Abbreviation	Full model name	Context window length (tokens)
LLAMA-3	Meta-Llama-3-8B-Instruct	8000
FLAN-T5	flan-t5-large	512
GEMMA-2	gemma-2-9b-it	8192
MISTRAL-0.2	Mistral-7B-Instruct-v0.2	32000
BIO-LLAMA-3	Bio-Medical-Llama-3-8B	8000

### Prompting Strategies for the ICL Approach

**Figure 2:**
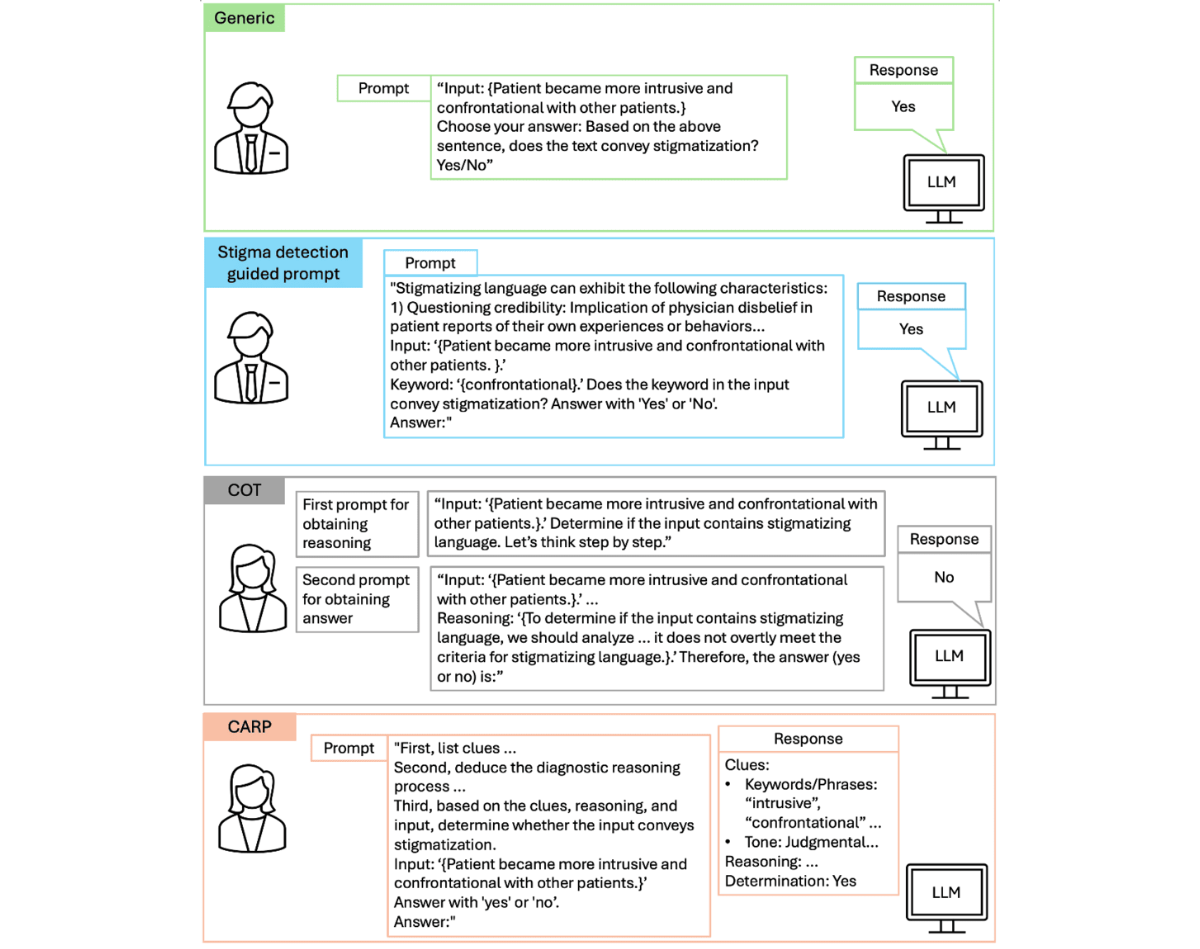
Examples of 4 distinct prompting strategies used in this study. CARP: clue and reasoning prompting; COT: chain of thought; LLM: large language model.

The effectiveness of the ICL approach relies heavily on the design of a well-structured prompting strategy that delivers clear and precise task instructions. A prompting strategy refers to the specific approach used to organize instructions, examples, and guiding principles within a prompt to elicit accurate and reliable outputs from the LLM. These prompts are often accompanied by either zero or a few illustrative examples to guide the model in performing downstream tasks effectively [29,32]. In this study, we evaluated 4 distinct prompting strategies: generic, COT [44], CARP [30], and a new customized prompting strategy we developed, referred to as the stigma detection guided prompt. The prompting template for each strategy is provided in Table 6. Figure 2 provides examples of the prompting strategies used in this study.

**Table 6:** Prompting strategies and templates used in the in-context learning approach.

Prompting strategy	Prompt template
Generic	“Input: ‘{input sentence}.’ Choose your answer: Based on the above sentence, does the text convey stigmatization? Yes/No”
Chain of thought	“Input: ‘{input sentence}.’ Determine if the input contains stigmatizing language. Let’s think step by step. Reasoning: ‘{reasoning}.’ Therefore, the answer (yes or no) is”:
Clue and reasoning prompting	“First, list clues (e.g., keywords, phrases, contextual information, semantic relations, tones, references) that support the determination of stigmatization in the input. Second, deduce the diagnostic reasoning process from the premises (i.e., clues and input) that support the stigmatization determination. Third, based on the clues, reasoning, and input, determine whether the input conveys stigmatization. Input: ‘{input sentence}.’ Answer with ‘yes’ or ‘no’. Answer:”
Stigma detection guided prompt	“Stigmatizing language can exhibit the following characteristics: 1) Questioning credibility: Implication of physician disbelief in patient reports of their own experiences or behaviors. 2) Disapproval: Highlights poor reasoning, decision-making, or self-care, often in a way that suggests the patient is unreasonable. 3) Stereotyping: Quoting incorrect grammar or unsophisticated terms. 4) Difficult patient: Inclusion of details with questionable clinical significance that portray the patient as belligerent or otherwise suggest that the physician is annoyed. 5) Unilateral decisions: Language that emphasizes physician authority. Input: ‘{input statement}.’ Keyword: “{keyword}.” Does the keyword in the input convey stigmatization? Answer with ‘Yes’ or ‘No’. Answer:”

The generic prompting strategy involves providing the model with minimal context, thereby relying primarily on its pretrained knowledge to perform the task [69]. In this approach, the prompt consists of a brief instruction about the task at hand, without incorporating reasoning steps or specific domain knowledge. For example, in the context of detecting stigmatizing language, the prompt might simply query the model with “Choose your answer: On the basis of the above sentence, does the text convey stigmatization? Yes/No.*”* This strategy serves as a baseline for comparing its performance with that of more advanced prompting techniques, such as COT, CARP, and the stigma detection guided prompt. In the few-shot scenario, a limited number of example sentences accompanied by their corresponding labels are embedded within the generic prompt to guide the model in making informed predictions.

The COT prompting strategy is designed to enhance the reasoning capabilities of general-purpose LLMs by guiding them to generate intermediate reasoning steps before arriving at a final prediction [44]. This approach leverages the LLM’s capacity to systematically reason through complex tasks, thereby improving the reliability and accuracy of its outputs. For instance, the prompt can be appended with “Let’s think step by step” to induce the model to break down the problem into logical and incremental steps. By explicitly instructing the model to articulate its reasoning, the COT strategy encourages more thoughtful predictions. In the zero-shot setting, the COT strategy involves prompting the model twice before making a prediction [33]. The first prompt encourages the model to produce intermediate reasoning by appending “Let’s think step by step” to the end of the prompt. This intermediate reasoning is then incorporated into a second prompt, which is used to generate the final prediction. In the few-shot setting, the model was first prompted to generate reasoning steps for randomly selected data points. These data points, along with their corresponding reasoning steps, were incorporated into the prompt as examples to enhance the model’s performance.

The CARP prompting strategy is specifically designed for text classification tasks. It uses a progressive reasoning approach to handle the linguistic complexities inherent in such tasks. This strategy first prompts the model to identify superficial clues within the input text, such as keywords, tone, semantic relations, and references, which provide initial indicators for classification. Once these clues are identified, the model engages in a diagnostic reasoning process to infer the final prediction in a logical manner [30]. For example, the presence of a negative tone combined with specific keywords may collectively suggest the presence of stigmatizing language. In the few-shot setting, the model was first guided to generate clues and reasoning for randomly selected examples. These examples, along with their associated clues, reasoning, and labels, were then incorporated into the prompt to enhance the model’s performance [30]. The detailed code implementation for the prompting strategies is provided in Multimedia Appendix 2.

### Design of the Stigma Detection Guided Prompt

In this study, we introduce a novel prompting strategy for detecting stigmatizing language, termed the stigma detection guided prompt. While prompt engineering has shown promise in improving LLM performance, there is no universally established framework for systematically designing prompts tailored to specific tasks [32]. The design of the proposed prompting strategy is inspired by recent research indicating that incorporating task-tailored instructions and domain knowledge can enhance the performance of the LLMs on downstream medical tasks [73,74]. Our proposed strategy consists of two key components: (1) explicitly directing the LLM to assess potentially stigmatizing words or phrases and (2) incorporating linguistic characteristics commonly associated with stigmatizing language to enhance the model’s ability to identify subtle instances of stigmatization in clinical text. In the subsequent section, we outline the rationale behind these components and their contributions to improving stigma detection in clinical documentation.

In the stigma detection guided prompt, we specifically instruct the LLM to assess potentially stigmatizing words or expressions. For instance, in the sentence “Patient became more intrusive and confrontational with other patients,” the model is specifically instructed to assess whether the term “confrontational” conveys stigmatization based on its surrounding context (Figure 2). We intentionally designed the prompt to focus on sentences containing expert-identified terms that are frequently used to convey stigmatization in clinical documentation (ie, aggressive and noncompliant). In this task formulation, the model is not asked to detect stigmatization broadly, but rather to assess whether the use of a known high-risk term conveys stigmatization in context. This reflects a practical and clinically relevant problem, as the same term may be neutral or stigmatizing depending on how it is used. In addition, this keyword-based approach aligns with prevailing practices in the literature. Previous studies have typically developed machine learning models or conducted analyses using sentences containing specific terms that are frequently flagged as stigmatizing according to medical language guidelines [15,75]. This approach remains widely adopted, in part because standardized definitions of stigmatizing language are still underdeveloped, and the topic remains sensitive within health care contexts. Therefore, focusing on predefined terms in the prompt offers a consistent and pragmatic approach for studying stigmatizing language across datasets and research settings. Conventional prompting strategies do not effectively leverage this information. By explicitly integrating this task-tailored instruction, our approach aims to enhance the LLM’s ability to detect stigmatizing language with greater contextual awareness.

Within the proposed prompting strategy, we also integrate linguistic characteristics commonly associated with stigmatizing language to enhance the model’s ability to detect subtle and implicit stigmatization. Stigmatizing language in EHRs frequently manifests in indirect ways, where certain words or phrases acquire negative connotations depending on the context in which they are used [19,76]. For instance, the term “claims” in the phrase “Patient claims pain is 10/10” may suggest disbelief, thereby contributing to stigma [7]. Previous work has identified five recurring linguistic patterns associated with stigmatizing language in clinical documentation, including (1) questioning patient credibility, (2) expressing disapproval of patient reasoning or self-care, (3) stereotyping based on race or social class, (4) portraying patients as difficult, and (5) emphasizing physician authority over the patient [37]. By incorporating these linguistic markers into the prompting strategy, our approach aims to enhance the LLM’s sensitivity to context-dependent cues, improving its overall accuracy in detecting stigmatizing language. In the few-shot setting, we incorporated examples within the prompt, following a similar approach to that used in the generic prompting strategy.

### Performance Evaluation and Comparison

In this study, stigmatizing language detection was framed as a binary text classification problem. To evaluate the performance of various approaches, we randomly sampled 1000 data points to create a test dataset, ensuring no overlap with training or validation datasets. We used 5 standard classification metrics: accuracy, precision, recall, area under the receiver operating characteristic curve (AUC-ROC), and *F*_1_-score. Accuracy indicates the percentage of EHR sentences that are correctly classified. Precision measures the proportion of sentences identified as stigmatizing that are stigmatizing, reflecting the model’s ability to avoid false positives. Recall, also known as sensitivity, assesses the proportion of actual stigmatizing sentences that are correctly identified, highlighting the model’s capacity to capture true positives. The AUC-ROC evaluates the model’s ability to distinguish between stigmatizing and nonstigmatizing sentences across different classification thresholds, with higher values indicating better discriminatory power. The *F*_1_-score, which ranges from 0 to 1, is computed as the harmonic mean of precision and recall, offering a balanced measure of classification performance. The *F*_1_-score is particularly critical for evaluating stigmatizing language detection due to the highly imbalanced nature of the dataset. Notably, the AUC-ROC metric was not computed for the ICL approach, as it does not produce the probabilistic classification required for the metric.

To obtain a robust estimate of the model’s performance, we computed 95% CIs by training, tuning, and evaluating each model on 10 different train-validation-test splits, each generated using a different random seed. These test datasets were designed to have no overlap with the training or validation datasets, thereby minimizing potential biases in performance estimation. To compare the top-performing models, we conducted paired 1-tailed *t* tests on *F*_1_-score, precision, and recall. Because each model was evaluated on the same test splits, the paired *t* test was chosen as it accounts for the dependency between paired observations, reducing variance and increasing statistical power compared to an independent *t* test. In the zero-shot setting, we compared the highest-performing models based on *F*_1_-score in both the textual entailment and ICL approaches. In the few-shot setting, we compared the best models when different numbers of annotations were available in the SetFit and ICL approaches. In addition, we compared the top-performing supervised fine-tuning model with the best ICL model. To mitigate the risk of inflated type I error due to multiple comparisons, we applied the Bonferroni correction to adjust the significance threshold. Given that a total of 15 comparisons were conducted, the adjusted significance level was set at *P*=.003 (.05/15).

### Fairness Evaluation

The notion of machine learning fairness in health care can be broadly classified into equal allocation and equal performance considerations [39]. Equal allocation pertains to the equitable distribution of health care resources across different patient subgroups. For instance, in the context of kidney transplantation, a fair machine learning model would ensure that transplants are allocated proportionally among patient groups, thereby mitigating disparities in access to care [77]. In contrast, equal performance requires that a model achieves comparable predictive accuracy across both protected and nonprotected groups. This can be quantified using metrics such as the true positive rate (TPR) and false positive rate (FPR), among others [39-41]. In the context of stigmatizing language detection, the equal performance criterion is more suitable as it ensures that the model identifies stigmatizing language consistently across different demographic groups.

For the fairness evaluation, we used the criterion of equal performance, assessing the model across 3 key performance metrics: TPR, FPR, and *F*_1_-score. A classifier is considered fair under this criterion if its performance remains consistent across subgroups defined by protected attributes. The TPR, also commonly referred to as recall, quantifies the proportion of EHR sentences containing stigmatizing language that the model correctly identifies. This metric is critical for fairness evaluation, as a lower TPR for certain demographic subgroups means the model fails to detect stigmatizing language in their clinical records at the same rate as others, potentially allowing harmful biases to persist unnoticed in medical documentation. This could lead to disparities in patient advocacy efforts, as undetected stigmatizing language may influence health care provider perceptions and contribute to differences in the quality of care received. Over time, systematic underdetection of stigmatizing language in specific groups may reinforce existing biases in health care systems, further marginalizing vulnerable populations. FPR quantifies the proportion of nonstigmatizing sentences that are incorrectly classified as stigmatizing. This metric is essential for fairness evaluation, as an elevated FPR for certain demographic subgroups indicates that the model is more likely to incorrectly classify nonstigmatizing language as stigmatizing for those groups, which could lead to unnecessary scrutiny of their clinical documentation. This overflagging may cause health care providers to become more hesitant in describing patient conditions accurately, potentially leading to vague or incomplete medical records. As a result, the quality of clinical communication may be compromised, which could negatively impact care coordination, patient trust, and clinical decision-making. Finally, the *F*_1_-score provides a comprehensive measure of the model’s classification performance by balancing precision and recall. Differences in *F*_1_-score across demographic subgroups indicate variability in the model’s utility—its effectiveness in balancing the detection (recall) and accuracy (precision) of stigmatizing language. A lower *F*_1_-score for certain groups suggests that the model is either failing to detect stigmatizing language when it should (low recall) or flagging nonstigmatizing language incorrectly (low precision). This inconsistency can undermine the trust and usability of the model in clinical decision-making, potentially leading to biased documentation practices and uneven policy enforcement, ultimately reducing the overall fairness and effectiveness of the system.

We assessed the fairness of the models concerning 3 protected attributes: sex (male and female); age (0-25 y, 26-50 y, 51-75 y, and >75 y); and race (White, Black, Hispanic, Asian, and other or unknown). We adopted age groupings similar to those used in previous studies examining the fairness of machine learning classifiers [40,41]. Bias at the subgroup level was quantified by measuring performance disparities across demographic subgroups [41,78]. For sex-differentiated groups, performance disparity was calculated as the difference in the model’s performance between one sex group and its complementary counterpart. For race- and age-differentiated groups, performance disparity was determined by the difference between the performance of a specific subgroup and the median performance across all subgroups within that attribute. A high-performance disparity indicates significant variation in the model’s performance across different groups, suggesting a higher level of unfairness. We measured performance disparity for subgroups defined by sex, age, and race and reported the highest absolute performance disparity for each demographic attribute across models using ICL and supervised fine-tuning approaches.

## Results

### Overview

We conducted a comprehensive evaluation of various approaches for detecting stigmatizing language in data-scarce settings. First, we assessed the effectiveness of textual entailment and ICL approaches within a zero-shot setting. Then, we evaluated the SetFit and ICL approaches in few-shot settings, using 4, 8, and 16 annotated data points per class. We compared supervised fine-tuning with ICL to assess how well ICL can perform with limited data compared to an established method that relies on a large amount of annotated data. This section presents the comparative performance of each approach in detecting stigmatizing language.

### Performance Evaluation for Zero-Shot Approaches

Table 7 presents the performance metrics of the various pretrained language models using the textual entailment approach under the zero-shot setting. Among the models evaluated, DEBERTA-M achieved the highest mean *F*_1_-score and accuracy of 0.723 (95% CI 0.718-0.728) and 73.8% (95% CI 73.2%-74.4%), respectively. Conversely, BERT-M exhibited the lowest performance, with a mean *F*_1_-score and accuracy of 0.615 (95% CI 0.606-0.624) and 64.2% (95% CI 63.8%-64.6%), respectively.

The performance of the various general-purpose LLMs using the zero-shot ICL approach is presented in Table 8. The stigma detection guided prompt strategy yielded the highest mean accuracy of 80.7% and a mean *F*_1_-score of 0.838 across all evaluated prompting strategies. Conversely, the generic prompting strategy demonstrated the lowest performance, with a mean accuracy of 69.4% and a mean *F*_1_-score of 0.746. The mean accuracy across the prompting strategies varied by 11.3 percentage points, and the mean *F*_1_-scores differed by 0.092, indicating a significant variability in performance based on the prompting strategy used. The stigma detection guided prompt paired with GEMMA-2 achieved the highest performance, with a mean accuracy of 83.5% (95% CI 82.9%-84.1%) and a mean *F*_1_-score of 0.858 (95% CI 0.854-0.862). The zero-shot ICL approach demonstrated superior performance metrics compared to the textual entailment approach. Specifically, the highest mean *F*_1_-score in the ICL approach, achieved by GEMMA-2, was 0.858, exceeding the best mean *F*_1_-score of 0.723 obtained by DEBERTA-M in the textual entailment approach by 0.135, representing an 18.7% improvement. A paired *t* test demonstrated that GEMMA-2 significantly outperformed DEBERTA-M in terms of mean *F*_1_-score, precision, and recall (*P*<.001). The detailed statistical results are provided in Multimedia Appendix 3. In addition, 3 out of 4 prompting strategies within the ICL approach resulted in higher mean accuracy and *F*_1_-scores than those observed with the textual entailment method.

**Table 7:** Performance of pretrained language models using the textual entailment approach.

Pretrained language models	Accuracy (%), mean (SD)	*F*_1_-score, mean (SD)	Precision, mean (SD)	Recall, mean (SD)	AUC-ROC^a^, mean (SD)
ROBERTA-M	72.9 (0.7)	0.713 (0.008)	0.698 (0.007)	0.729 (0.008)	0.753 (0.009)
BART-M	69.6 (0.9)	0.691 (0.004)	0.701 (0.006)	0.681 (0.011)	0.736 (0.008)
DEBERTA-M	*73.8 (0.6)* ^b^	*0.723 (0.005)*	*0.717 (0.007)*	*0.730 (0.006)*	*0.781 (0.010)*
BERT-M	64.2 (0.4)	0.615 (0.009)	0.623 (0.008)	0.608 (0.010)	0.701 (0.007)
Overall	70.1 (0.7)	0.686 (0.006)	0.685 (0.007)	0.687 (0.009)	0.743 (0.008)

^a^AUC-ROC: area under the receiver operating characteristic curve.

^b^Italicized values represent the best performance across models.

**Table 8:** Performance of general-purpose large language models (LLMs) using the zero-shot in-context learning approach.

General-purpose LLM			Accuracy (%), mean (SD)		*F*_1_-score, mean (SD)		Precision, mean (SD)		Recall, mean (SD)
**Generic**									
	LLAMA-3	70.9 (0.8)		0.811 (0.005)		0.821 (0.006)		0.801 (0.004)	
	FLAN-T5	67.2 (0.6)		0.709 (0.007)		0.731 (0.008)		0.688 (0.005)	
	GEMMA-2	70.4 (0.9)		0.750 (0.004)		0.778 (0.004)		0.724 (0.004)	
	MISTRAL-0.2	68.4 (0.6)		0.705 (0.006)		0.713 (0.006)		0.698 (0.005)	
	BIO-LLAMA-3	70.1 (0.8)		0.762 (0.006)		0.774 (0.005)		0.750 (0.004)	
	Overall	69.4 (0.7)		0.746 (0.005)		0.763 (0.007)		0.732 (0.004)	
**Chain of thought**									
	LLAMA-3	74.5 (0.7)		0.781 (0.009)		0.792 (0.007)		0.771 (0.009)	
	FLAN-T5	73.6 (0.3)		0.768 (0.007)		0.779 (0.006)		0.757 (0.005)	
	GEMMA-2	75.2 (0.6)		0.795 (0.005)		0.813 (0.007)		0.777 (0.004)	
	MISTRAL-0.2	72.5 (0.4)		0.756 (0.008)		0.768 (0.004)		0.745 (0.009)	
	BIO-LLAMA-3	73.1 (0.7)		0.775 (0.006)		0.787 (0.007)		0.764 (0.009)	
	Overall	73.8 (0.5)		0.775 (0.007)		0.788 (0.006)		0.763 (0.007)	
**Clue and reasoning prompting**									
	LLAMA-3	78.3 (0.6)		0.830 (0.005)		*0.842 (0.004)^a^*		0.818 (0.005)	
	FLAN-T5	74.9 (0.7)		0.785 (0.008)		0.794 (0.008)		0.776 (0.007)	
	GEMMA-2	73.7 (0.9)		0.803 (0.010)		0.811 (0.005)		0.795 (0.009)	
	MISTRAL-0.2	70.8 (0.5)		0.736 (0.007)		0.752 (0.005)		0.721 (0.007)	
	BIO-LLAMA-3	77.9 (0.6)		0.815 (0.005)		0.831 (0.007)		0.799 (0.004)	
	Overall	75.1 (0.7)		0.794 (0.008)		0.806 (0.006)		0.782 (0.007)	
**Stigma detection guided prompt**									
	LLAMA-3	82.4 (0.6)		0.845 (0.005)		0.822 (0.006)		0.869 (0.008)	
	FLAN-T5	76.6 (0.8)		0.819 (0.005)		0.804 (0.004)		0.834 (0.007)	
	GEMMA-2	*83.5 (0.6)*		*0.858 (0.004)*		*0.841 (0.005)*		*0.876 (0.003)*	
	MISTRAL-0.2	80.1 (0.5)		0.830 (0.004)		0.813 (0.006)		0.847 (0.006)	
	BIO-LLAMA-3	81.0 (0.5)		0.839 (0.005)		0.857 (0.007)		0.821 (0.004)	
	Overall	80.7 (0.6)		0.838 (0.005)		0.827 (0.006)		0.849 (0.005)	

^a^Italicized values represent the best performance across models and prompting strategies.

### Performance Evaluation for Few-Shot Approaches

Table 9 presents the performance of the various pretrained sentence transformers using the SetFit approach in the few-shot setting across different numbers of labeled data points per class. MINILM-V2 achieved the highest mean *F*_1_-score of 0.721 (95% CI 0.713-0.729) and the highest mean AUC-ROC of 0.791 (95% CI 0.785- 0.797) with 4 annotated data points per class. However, E5-V2 outperformed the other models when 8 and 16 annotated data points per class were available, achieving mean *F*_1_-scores of 0.735 (95% CI 0.728-0.742) and 0.802 (95% CI 0.798-0.806), respectively. On average, accuracy increased from 72.1% with 4 annotated data points per class to 78% with 16 annotated data points per class, while the average *F*_1_-score improved from 0.707 to 0.767. These results highlight the positive impact of increasing the number of annotated data points on model performance within the few-shot SetFit approach.

Table 10 presents the mean *F*_1_-score and accuracy of various general-purpose LLMs using the few-shot ICL approach with different prompting strategies. Additional performance metrics, including precision and recall, are provided in Multimedia Appendix 4. Due to limitations in the number of tokens (or words) that models can process at once (known as context window limitation), it was not feasible to conduct experiments with FLAN-T5 for the COT and CARP prompting strategies in the few-shot settings, nor with LLAMA-3 and GEMMA-2 for COT and CARP when 16 annotated data points per class were used. Thus, we do not report the mean performance of COT and CARP prompting strategies across models. LLAMA-3, paired with the stigma detection guided prompt, demonstrated the highest performance across all scenarios, achieving mean *F*_1_-scores of 0.874 (95% CI 0.864-0.884), 0.892 (95% CI 0.886-0.898), and 0.901 (95% CI 0.895-0.907) with 4, 8, and 16 annotated data points per class, respectively. When using the generic and the stigma detection guided prompt, it was observed that both the mean *F*_1_-score and accuracy improved as the number of annotated data points per class increased.

**Table 9:** Performance of pretrained sentence transformers using the SetFit approach.

Sentence transformer		Accuracy (%), mean (SD)	*F*_1_-score, mean (SD)	Precision, mean (SD)	Recall, mean (SD)	AUC-ROC^a^, mean (SD)
**4 annotated data per class**						
	MPNET-V2	70.5 (0.4)	0.701 (0.005)	0.708 (0.007)	0.697 (0.005)	0.752 (0.005)
	E5-V2	*73.6 (0.6)* ^b^	0.704 (0.003)	0.705 (0.004)	0.703 (0.003)	0.784 (0.007)
	ROBERTA-V2	72.2 (0.6)	0.708 (0.004)	0.712 (0.005)	0.704 (0.005)	0.765 (0.005)
	MINILM-V2	73.0 (0.5)	*0.721 (0.008)*	*0.718 (0.007)*	*0.725 (0.005)*	*0.791 (0.006)*
	BERT-BIO-ST	71.4 (0.7)	0.701 (0.008)	0.687 (0.004)	0.715 (0.006)	0.770 (0.008)
	BIOLORD	72.0 (0.7)	0.707 (0.008)	0.714 (0.005)	0.701 (0.009)	0.772 (0.005)
	Overall	72.1 (0.6)	0.707 (0.006)	0.706 (0.006)	0.707 (0.005)	0.772 (0.006)
**8 annotated data per class**						
	MPNET-V2	72.6 (0.8)	0.715 (0.003)	0.727 (0.006)	0.704 (0.004)	0.773 (0.005)
	E5-V2	*77.1 (0.4)*	*0.735 (0.007)*	*0.750 (0.006)*	0.721 (0.008)	*0.836 (0.006)*
	ROBERTA-V2	75.4 (0.8)	0.723 (0.005)	0.729 (0.006)	0.718 (0.004)	0.814 (0.006)
	MINILM-V2	74.3 (0.6)	0.732 (0.005)	0.721 (0.004)	*0.743 (0.005)*	0.801 (0.007)
	BERT-BIO-ST	75.1 (0.7)	0.726 (0.007)	0.715 (0.008)	0.736 (0.005)	0.817 (0.005)
	BIOLORD	75.9 (0.4)	0.729 (0.008)	0.718 (0.005)	0.740 (0.007)	0.821 (0.004)
	Overall	75.1 (0.5)	0.727 (0.006)	0.726 (0.007)	0.728 (0.006)	0.810 (0.005)
**16 annotated data per class**						
	MPNET-V2	75.6 (0.3)	0.755 (0.005)	0.773 (0.005)	0.738 (0.006)	0.815 (0.007)
	E5-V2	*80.7 (0.5)*	*0.802 (0.004)*	*0.813 (0.006)*	*0.792 (0.003)*	*0.858 (0.006)*
	ROBERTA-V2	78.3 (0.4)	0.775 (0.006)	0.766 (0.007)	0.784 (0.004)	0.838 (0.005)
	MINILM-V2	79.1 (0.6)	0.785 (0.007)	0.802 (0.006)	0.769 (0.005)	0.847 (0.009)
	BERT-BIO-ST	77.3 (0.5)	0.745 (0.008)	0.752 (0.007)	0.738 (0.008)	0.832 (0.004)
	BIOLORD	77.1 (0.8)	0.739 (0.004)	0.744 (0.005)	0.734 (0.004)	0.829 (0.006)
	Overall	78.0 (0.7)	0.767 (0.006)	0.775 (0.005)	0.759 (0.007)	0.837 (0.007)

^a^AUC-ROC: area under the receiver operating characteristic curve.

^b^Italicized values represent the best performance across models when different numbers of annotated data per class were available.

Table 11 presents a comparison between the top-performing models using the SetFit approach and those using the few-shot ICL approach across different numbers of annotated data points per class. In all scenarios, the few-shot ICL models exhibited superior performance compared to their SetFit counterparts. Specifically, with 4 annotated data points per class, LLAMA-3 achieved a mean *F*_1_-score of 0.874 (95% CI 0.864-0.884), which is 0.153 points higher than that of MINILM-V2, whose mean *F*_1_-score was 0.721 (95% CI 0.713-0.729), representing an approximate 21.2% improvement. For 8 annotated data points per class, LLAMA-3 attained a mean *F*_1_-score of 0.892 (95% CI 0.886-0.898) compared to 0.735 (95% CI 0.728-0.742) for E5-V2, corresponding to an improvement of about 21.4%. Finally, at 16 annotated data points per class, LLAMA-3 demonstrated a mean *F*_1_-score of 0.901 (95% CI 0.895-0.907), which is 0.099 points higher than that of E5-V2, whose mean *F*_1_-score was 0.802 (95% CI 0.798-0.806), reflecting a 12.3% improvement. For each setting (4,8, and 16 annotated data points per class), a paired *t* test was conducted to compare the top-performing models from the ICL and SetFit approaches (refer to Multimedia Appendix 3 for details). In all cases, the results indicated that the ICL models outperformed the SetFit models in terms of *F*_1_-score, precision, and recall (*P*<.001).

**Table 10:** Performance of general-purpose large language models (LLMs) using the few-shot in-context learning approach.

General purpose-LLM		4 annotated data per class			8 annotated data per class			16 annotated data per class	
		Accuracy (%), mean (SD)	*F*_1_-score, mean (SD)	Accuracy (%), mean (SD)		*F*_1_-score, mean (SD)	Accuracy (%), mean (SD)		*F*_1_-score, mean (SD)
**Generic**									
	LLAMA-3	74.8 (0.8)	0.789 (0.006)	79.2 (0.9)		0.832 (0.008)	81.6 (0.8)		0.855 (0.006)
	FLAN-T5	69.3 (0.5)	0.691 (0.007)	70.7 (0.8)		0.701 (0.008)	75.1 (0.5)		0.762 (0.006)
	GEMMA-2	71.1 (1.2)	0.719 (0.007)	72.1 (0.8)		0.739 (0.009)	76.2 (0.9)		0.773 (0.008)
	MISTRAL-0.2	72.5 (0.7)	0.730 (0.006)	70.5 (0.9)		0.720 (0.010)	80.1 (1.0)		0.822 (0.007)
	BIO-LLAMA-3	76.6 (0.9)	0.820 (0.008)	77.3 (0.5)		0.826 (0.004)	79.0 (0.6)		0.851 (0.006)
	Overall	72.9 (0.8)	0.75 (0.007)	74.0 (0.8)		0.764 (0.009)	78.4 (0.6)		0.813 (0.007)
**COT**									
	LLAMA-3	72.5 (0.8)	0.756 (0.004)	83.1 (0.9)		0.823 (0.007)	—^a^		—
	FLAN-T5	—	—	—		—	—		—
	GEMMA-2	75.1 (0.4)	0.796 (0.006)	79.5 (0.8)		0.826 (0.005)	—		—
	MISTRAL-0.2	71.3 (0.3)	0.742 (0.005)	75.1 (0.6)		0.767 (0.007)	76.9 (0.8)		0.781 (0.006)
	BIO-LLAMA-3	71.6 (0.7)	0.743 (0.005)	81.4 (0.8)		0.835 (0.006)	—		—
**CARP**									
	LLAMA-3	80.4 (0.8)	0.837 (0.005)	85.1 (0.5)		0.876 (0.004)	—		—
	FLAN-T5	—	—	—		—	—		—
	GEMMA-2	75.4 (0.7)	0.795 (0.006)	79.6 (0.4)		0.825 (0.009)	—		—
	MISTRAL-0.2	71.8 (0.5)	0.728 (0.008)	72.2 (0.5)		0.747 (0.006)	71.2 (0.6)		0.731 (0.005)
	BIO-LLAMA-3	77.2 (0.7)	0.813 (0.009)	81.6 (0.6)		0.849 (0.008)	—		—
**Stigma detection guided prompt**									
	LLAMA-3	*84.3 (0.6)* ^b^	*0.874 (0.010)*	*85.5 (0.7)*		*0.892 (0.006)*	*87.7 (0.7)*		*0.901 (0.006)*
	FLAN-T5	74.2 (0.4)	0.782 (0.006)	76.8 (0.5)		0.805 (0.008)	82.8 (0.3)		0.842 (0.004)
	GEMMA-2	84.3 (0.9)	0.872 (0.007)	84.9 (0.4)		0.881 (0.005)	86.1 (0.5)		0.887 (0.006)
	MISTRAL-0.2	80.5 (0.7)	0.849 (0.005)	82.0 (0.6)		0.857 (0.004)	86.7 (0.7)		0.899 (0.005)
	BIO-LLAMA-3	80.4 (1.1)	0.861 (0.010)	81.5 (0.8)		0.870 (0.007)	82.7 (1.0)		0.892 (0.009)
	Overall	80.7 (0.8)	0.848 (0.008)	82.1 (0.6)		0.861 (0.005)	85.2 (0.5)		0.884 (0.007)

^a^Not applicable due to limited context window length.

^b^Italicized values represent the best performance across models and prompting strategies when different amounts of annotated data per class were available.

**Table 11:** Comparison of top-performing models in SetFit and few-shot in-context learning (ICL) approaches.

Approach and model			4 annotated data per class—*F*_1_-score, mean (SD)		8 annotated data per class—*F*_1_-score, mean (SD)		16 annotated data per class—*F*_1_-score, mean (SD)
**SetFit**							
	MINILM-V2	0.721 (0.008)		—^a^		—	
	E5-V2	—		0.735 (0.007)		0.802 (0.004)	
**Few-shot ICL**							
	LLAMA-3	0.874 (0.010)		0.892 (0.006)		0.901 (0.006)	

^a^Not applicable.

### Performance Comparison of the ICL With the Fully Supervised Fine-Tuning Approach

We presented the performance of the various pretrained language models using the fully supervised fine-tuning approach in Table 12. Among the models evaluated, ROBERTA demonstrated the highest performance, achieving a mean AUC-ROC of 0.973 (95% CI 0.968-0.978) and a mean *F*_1_-score of 0.931 (95% CI 0.924-0.938). In contrast, BERT-BIOMED exhibited the lowest performance, with a mean AUC-ROC of 0.926 (95% CI 0.918-0.934) and a mean *F*_1_-score of 0.894 (95% CI 0.888-0.900).

**Table 12:** Performance of pretrained language models for the fully supervised fine-tuning approach.

Pretrained language model	Accuracy (%), mean (SD)	*F*_1_-score, mean (SD)	Precision, mean (SD)	Recall, mean (SD)	AUC-ROC^a^, mean (SD)
ROBERTA	*94.5 (0.6)* ^b^	*0.931 (0.007)*	*0.950 (0.008)*	*0.913 (0.009)*	*0.973 (0.005)*
BERT	94.2 (0.7)	0.922 (0.005)	0.943 (0.007)	0.901 (0.011)	0.962 (0.004)
BERT-BIO	93.7 (0.8)	0.910 (0.009)	0.929 (0.006)	0.893 (0.006)	0.948 (0.003)
ROBERTA-XLM	94.0 (0.5)	0.911 (0.006)	0.945 (0.007)	0.879 (0.008)	0.943 (0.009)
BERT-BIOMED	91.5 (0.7)	0.894 (0.006)	0.909 (0.004)	0.879 (0.008)	0.926 (0.008)
Overall	93.6 (0.6)	0.914 (0.007)	0.935 (0.007)	0.893 (0.008)	0.950 (0.006)

^a^AUC-ROC: area under the receiver operating characteristic curve.

^b^Italicized values represent the best performance across models.

Table 13 presents the performance of the top-performing models in the zero-shot, few-shot settings, and fully supervised fine-tuning approach. In the zero-shot setting, the best-performing model, GEMMA-2, achieved a mean *F*_1_-score of 0.858 (95% CI 0.854-0.862), which is 0.073 points lower than the mean *F*_1_-score of fully supervised ROBERTA at 0.931 (95% CI 0.924-0.938). This difference corresponds to an approximate 8.5% improvement in mean *F*_1_-score for the fully supervised approach. In the few-shot setting, LLAMA-3 emerged as the top-performing model, achieving mean *F*_1_-scores of 0.874 (95% CI 0.864-0.884), 0.892 (95% CI 0.886-0.898), and 0.901 (95% CI 0.895-0.907) with 4, 8, and 16 annotated data points per class, respectively. Although the mean *F*_1_-score with 16 annotated data points per class was about 3.2% lower than that of fully supervised ROBERTA, it is notable that this performance was attained using only 32 labeled instances, whereas ROBERTA was trained with 3543 instances—more than 100 times the amount of annotated data.

**Table 13:** Performance of top-performing models in zero-shot, few-shot, and fully supervised fine-tuning approaches.

Approach or model	GEMMA-2—*F*_1_-score, mean (SD)	LLAMA-3—*F*_1_-score, mean (SD)	ROBERTA—*F*_1_-score, mean (SD)
Zero-shot	0.858 (0.004)	—^a^	—
Few-shot: 4 annotated data per class	—	0.874 (0.010)	—
Few-shot: 8 annotated data per class	—	0.892 (0.006)	—
Few-shot: 16 annotated data per class	—	0.901 (0.006)	—
Fully supervised fine-tuning	—	—	0.931 (0.007)

^a^Not applicable.

### Statistical Comparison of Model Performance

Table 14 summarizes the key findings from paired *t* tests, comparing the top-performing models in the zero-shot and few-shot settings, as well as performance differences between the best-performing ICL and supervised fine-tuning models. Additional details on the statistical comparisons are provided in Multimedia Appendix 3. In the zero-shot setting, paired *t* tests revealed that GEMMA-2 significantly outperformed DEBERTA-M in terms of *F*_1_-score, precision, and recall (all *P*<.001). In the few-shot setting, ICL models demonstrated significantly higher performance across all 3 metrics compared to SetFit models. When comparing the top-performing models from the ICL and supervised fine-tuning approaches, ROBERTA achieved significantly higher *F*_1_-score and precision (*P*<.001), while LLAMA-3 demonstrated significantly higher recall (*P*<.001).

**Table 14:** Paired *t* test results comparing the top-performing models in the zero-shot and few-shot settings, and between the best-performing models in in-context learning and supervised fine-tuning approaches.

Setting, comparison, and metric		Mean difference	*P* value
**Zero-shot: DEBERTA-M versus GEMMA-2 coupled with stigma detection guided prompt**			
	*F*_1_-score	−0.136	<.001
	Precision	−0.125	<.001
	Recall	−0.146	<.001
**Few-shot (4 annotations per label): MINILM-V2 versus LLAMA 3 coupled with stigma detection guided prompt**			
	*F*_1_-score	−0.155	<.001
	Precision	−0.138	<.001
	Recall	−0.165	<.001
**Few-shot (8 annotations per label): E5-V2 versus LLAMA 3 coupled with stigma detection guided prompt**			
	*F*_1_-score	−0.158	<.001
	Precision	−0.124	<.001
	Recall	−0.192	<.001
**Few-shot (16 annotations per label): E5-V2 versus LLAMA 3 coupled with stigma detection guided prompt**			
	*F*_*1*_-score	−0.101	<.001
	Precision	−0.052	<.001
	Recall	−0.152	<.001
**ICL versus supervised fine-tuning: ROBERTA versus LAMMA-3 coupled with stigma detection guided prompt (16 annotated data per class)**			
	*F*_1_-score	0.029	<.001
	Precision	0.086	<.001
	Recall	−0.030	<.001

### Fairness Evaluation and Comparison

The performance and disparities of various models developed to detect stigmatizing language are presented in Multimedia Appendix 5. Table 15 highlights the largest absolute performance disparities across demographic attributes, including sex, age, and race, for each model. Models using the supervised fine-tuning approach exhibited relatively higher absolute disparities compared to the ICL models under zero-shot, 4-shot, 8-shot, and 16-shot settings. On average, models trained using the supervised fine-tuning approach demonstrated the largest mean absolute TPR disparities of 0.037, 0.077, and 0.046 for sex-, age-, and race-differentiated groups, respectively. These disparities indicate that the TPR can differ by up to 7.7% in certain demographic subgroups. In contrast, ICL models showed a more balanced TPR, with no mean disparity exceeding 0.007, 0.010, and 0.013 for sex-, age-, and race-differentiated groups, respectively. A similar pattern was observed for FPR and *F*_1_-scores. Supervised fine-tuning models exhibited greater FPR disparities, with mean values of 0.026, 0.027, and 0.031 for sex-, age-, and race-differentiated groups, respectively. Conversely, the ICL models demonstrated lower FPR disparities under different experimental settings, indicating a more consistent FPR across groups. Table 16 presents the highest performance disparities of ICL and supervised fine-tuning models across sex, age, and race subgroups. Here, the highest disparities refer to the highest observed differences in performance metrics across demographic groups for each modeling approach. For TPR, supervised models exhibited disparities of up to 0.051 for sex, 0.108 for age, and 0.064 for race. In contrast, both zero-shot and few-shot ICL models showed substantially lower TPR disparities, with all values less than 0.016. Similarly, FPR disparities reached up to 0.039 for sex, 0.037 for age, and 0.043 for race in supervised models, whereas FPR disparities for ICL models remained less than 0.027. Regarding *F*_1_-scores, the highest disparity among supervised models reached 0.086, compared to less than 0.025 among ICL models. These findings suggest that ICL models offer more equitable performance across demographic groups.

**Table 15:** The largest absolute performance disparity across demographic attributes for different approaches in detecting stigmatizing language.

Approach and model				Sex		Age (y)		Race
**Largest absolute TPR^a^disparity**								
	**Supervised fine-tuning**							
		ROBERTA	0.049		0.108		0.044	
		BERT	0.051		0.058		0.036	
		BERT-BIO	0.022		0.075		0.037	
		ROBERTA-XLM	0.033		0.089		0.052	
		BERT-BIOMED	0.032		0.053		0.064	
		Overall	0.037		0.077		0.046	
	**Zero-shot ICL^b^**							
		LLAMA-3	0.005		0.012		0.011	
		FLAN-T5	0.002		0.004		0.008	
		GEMMA-2	0.003		0.009		0.010	
		MISTRAL-0.2	0.007		0.005		0.006	
		BIO-LLAMA-3	0.006		0.010		0.013	
		Overall	0.005		0.008		0.010	
	**Few-shot ICL (4 annotated data per class)**							
		LLAMA-3	0.003		0.008		0.013	
		FLAN-T5	0.004		0.009		0.014	
		GEMMA-2	0.002		0.007		0.012	
		MISTRAL-0.2	0.014		0.006		0.015	
		BIO-LLAMA-3	0.008		0.008		0.010	
		Overall	0.006		0.008		0.013	
	**Few-shot ICL (8 annotated data per class)**							
		LLAMA-3	0.006		0.008		0.010	
		FLAN-T5	0.003		0.005		0.009	
		GEMMA-2	0.006		0.007		0.011	
		MISTRAL-0.2	0.014		0.004		0.007	
		BIO-LLAMA-3	0.005		0.008		0.007	
		Overall	0.007		0.006		0.009	
	**Few-shot ICL (16 annotated data per class)**							
		LLAMA-3	0.003		0.007		0.012	
		FLAN-T5	0.007		0.008		0.007	
		GEMMA-2	0.006		0.012		0.013	
		MISTRAL-0.2	0.007		0.011		0.009	
		BIO-LLAMA-3	0.006		0.013		0.010	
		Overall	0.006		0.010		0.010	
**Largest absolute FPR^c^ disparity**								
	**Supervised fine-tuning**							
		ROBERTA	0.018		0.022		0.028	
		BERT	0.016		0.015		0.023	
		BERT-BIO	0.035		0.033		0.027	
		ROBERTA-XLM	0.021		0.023		0.043	
		BERT-BIOMED	0.039		0.037		0.032	
		Overall	0.026		0.027		0.031	
	**Zero-shot ICL**							
		LLAMA-3	0.003		0.008		0.008	
		FLAN-T5	0.007		0.011		0.010	
		GEMMA-2	0.008		0.018		0.019	
		MISTRAL-0.2	0.005		0.016		0.014	
		BIO-LLAMA-3	0.001		0.011		0.008	
		Overall	0.005		0.013		0.012	
	**Few-shot ICL (4 annotated data per class)**							
		LLAMA-3	0.006		0.013		0.007	
		FLAN-T5	0.007		0.015		0.011	
		GEMMA-2	0.005		0.013		0.015	
		MISTRAL-0.2	0.003		0.016		0.014	
		BIO-LLAMA-3	0.005		0.017		0.015	
		Overall	0.005		0.015		0.012	
	**Few-shot ICL (8 annotated data per class)**							
		LLAMA-3	0.002		0.007		0.009	
		FLAN-T5	0.011		0.026		0.019	
		GEMMA-2	0.012		0.013		0.010	
		MISTRAL-0.2	0.007		0.021		0.014	
		BIO-LLAMA-3	0.005		0.011		0.014	
		Overall	0.007		0.016		0.013	
	**Few-shot ICL (16 annotated data per class)**							
		LLAMA-3	0.007		0.015		0.012	
		FLAN-T5	0.009		0.011		0.009	
		GEMMA-2	0.004		0.013		0.014	
		MISTRAL-0.2	0.004		0.016		0.013	
		BIO-LLAMA-3	0.014		0.017		0.010	
		Overall	0.008		0.014		0.011	
**Largest absolute** ***F***_**1**_ **-score disparity**								
	**Supervised fine-tuning**							
		ROBERTA	0.043		0.066		0.064	
		BERT	0.037		0.048		0.030	
		BERT-BIO	0.026		0.058		0.031	
		ROBERTA-XLM	0.064		0.086		0.032	
		BERT-BIOMED	0.041		0.053		0.051	
		Overall	0.042		0.063		0.042	
	**Zero-shot ICL**							
		LLAMA-3	0.006		0.015		0.006	
		FLAN-T5	0.004		0.018		0.011	
		GEMMA-2	0.010		0.013		0.016	
		MISTRAL-0.2	0.006		0.015		0.010	
		BIO-LLAMA-3	0.005		0.005		0.009	
		Overall	0.006		0.013		0.010	
	**Few-shot ICL (4 annotated data per class)**							
		LLAMA-3	0.005		0.010		0.017	
		FLAN-T5	0.012		0.011		0.013	
		GEMMA-2	0.008		0.013		0.014	
		MISTRAL-0.2	0.004		0.016		0.013	
		BIO-LLAMA-3	0.010		0.011		0.018	
		Overall	0.008		0.012		0.015	
	**Few-shot ICL (8 annotated data per class)**							
		LLAMA-3	0.008		0.009		0.006	
		FLAN-T5	0.010		0.023		0.016	
		GEMMA-2	0.003		0.013		0.015	
		MISTRAL-0.2	0.018		0.017		0.024	
		BIO-LLAMA-3	0.015		0.012		0.019	
		Overall	0.011		0.015		0.016	
	**Few-shot ICL (16 annotated data per class)**							
		LLAMA-3	0.006		0.014		0.014	
		FLAN-T5	0.013		0.020		0.025	
		GEMMA-2	0.008		0.011		0.017	
		MISTRAL-0.2	0.015		0.014		0.018	
		BIO-LLAMA-3	0.005		0.013		0.011	
		Overall	0.009		0.014		0.017	

^a^TPR: true positive rate.

^b^ICL: in-context learning.

^c^FPR: false positive rate.

**Table 16:** The highest observed performance disparities of in-context learning (ICL) and supervised fine-tuning models across sex, age, and race subgroups.

Approach		Sex	Age (y)	Race
**Highest TPR^a^ disparity**				
	Supervised fine-tuning	0.051	0.108	0.064
	Zero-shot ICL	0.007	0.012	0.013
	Few-shot ICL	0.014	0.013	0.015
**Highest FPR^b^ disparity**				
	Supervised fine-tuning	0.039	0.037	0.043
	Zero-shot ICL	0.008	0.018	0.019
	Few-shot ICL	0.014	0.026	0.019
**Highest *F*_1_-score disparity**				
	Supervised fine-tuning	0.064	0.086	0.064
	Zero-shot ICL	0.010	0.018	0.016
	Few-shot ICL	0.018	0.023	0.025

^a^TPR: true positive rate.

^b^FPR: false positive rate.

## Discussion

### Overview

The prevalence of stigmatizing language in EHRs has raised significant concern due to its detrimental impact on the quality of care [7,15,22,24,26,79]. Numerous studies have investigated the potential of using supervised machine learning approaches for detecting stigmatizing language [15,27,28]. However, these approaches typically require extensive amounts of labeled data to achieve optimal performance. The creation of such a dataset is particularly challenging, given the difficulty in identifying stigmatizing language, which is often subtle, implicit, and highly contextual [27,37]. The ICL approach has emerged as a promising alternative due to its strong contextual understanding capabilities and minimal reliance on labeled data [29-32]. In this study, we extensively investigated the efficacy of ICL in detecting stigmatizing language and compared its performance with established zero-shot and few-shot text classification methods. To the best of our knowledge, this is the first study to explore the use of ICL, as well as zero-shot and few-shot approaches, for detecting stigmatizing language. Furthermore, we proposed a novel prompting strategy designed for detecting stigmatizing language and evaluated its effectiveness against state-of-the-art prompting strategies, such as COT and CARP.

### Principal Findings

The ICL approaches have demonstrated a substantial advantage in detecting stigmatizing language compared to both the popular zero-shot textual entailment approach and the few-shot SetFit approach. In the zero-shot setting, the top-performing ICL model, GEMMA-2, achieved a mean *F*_1_-score of 0.858 (95% CI 0.854-0.862), surpassing the best mean *F*_1_-score of 0.723 (95% CI 0.718-0.728) obtained by ROBERTA-M in the textual entailment approach by 0.135 points, representing an 18.7% improvement. Similarly, in the few-shot setting, the top-performing ICL model, LLAMA-3, exhibited mean *F*_1_-score improvements of 21.2%, 21.4%, and 12.3% over the leading SetFit models when using 4, 8, and 16 annotations per class, respectively. The superior performance of the ICL approach can be attributed to the enhanced contextual understanding capability and rich knowledge of general-purpose LLMs [33,34]. These general-purpose LLMs have undergone sophisticated training processes, such as instructional fine-tuning, reinforcement learning from human feedback, and extensive pretraining across a diverse range of NLP tasks, including language modeling, text completion, and semantic understanding on vast amounts of textual data [66,80,81]. These training processes have enabled the general-purpose models to better comprehend and contextualize subtle and complex linguistic patterns, making the ICL approach particularly effective for detecting stigmatizing language in EHRs. Therefore, in settings where annotated data is limited, the ICL approach should be prioritized for detecting stigmatizing language. However, it is important to note that the ICL models do not produce probabilistic classification outputs, which prevents the computation of threshold-independent metrics, such as AUC-ROC. This limitation affects the interpretation of results in 2 key ways. First, it may obscure comparisons with probabilistic models, which may exhibit strong overall class separation but suffer from performance instability at specific thresholds (ie, precision, recall, or *F*_1_-scores). Second, more importantly, the lack of probability outputs prevents us from adjusting the decision threshold to accommodate different preferences for false positives versus false negatives, which is a common requirement in some clinical applications. Some clinical contexts may prioritize minimizing false positives to avoid overalerting, while others may emphasize minimizing false negatives to avoid missed harms.

Within the ICL approach, substantial performance variability was observed across different prompting strategies. Specifically, in the zero-shot setting, the mean accuracy for the ICL approach varied between 69.4% and 80.7% across different prompting strategies, indicating a gap of 11.3%. In the few-shot setting, the differences in mean accuracy across prompting strategies were 7.8%, 8.1%, and 6.8% when using 4, 8, and 16 annotations per class, respectively. These performance gaps highlight the critical importance of selecting an appropriate prompting strategy to optimize the effectiveness of the ICL approach in detecting stigmatizing language. The new prompting strategy that we proposed in this work, the stigma detection guided prompt, outperformed the established strategies, such as COT and CARP [30,44]. This strategy achieved the highest *F*_1_-scores across both zero-shot and few-shot settings, with a mean *F*_1_-score of 0.858 (95% CI 0.854-0.862) in the zero-shot setting and mean *F*_1_-scores of 0.874 (95% CI 0.864-0.884), 0.892 (95% CI 0.886-0.898), and 0.901 (95% CI 0.895-0.907) in the few-shot settings using 4, 8, and 16 annotations per class, respectively. The goal of the newly proposed prompt is to encode domain-specific knowledge that can assist the LLM in accurately identifying stigmatizing language. This was inspired by the fact that domain-specific information has been used in various settings to improve the performance of artificial intelligence (AI) algorithms, such as designing efficient search heuristics for informed search [82] or enhancing machine learning accuracy [83,84]. The prompt incorporated linguistic cues commonly associated with stigmatizing language, such as stereotyping, disapproval, and questioning credibility [37]. These elements enhance the model’s ability to detect nuanced stigmatizing language that might otherwise be overlooked. Consequently, the stigma detection guided prompt represents a critical advancement in optimizing the ICL approach for this task. Its demonstrated effectiveness underscores the importance of leveraging medical informatics–driven insights to improve the detection of stigmatizing language, thereby supporting bias-aware clinical documentation practices.

To thoroughly assess the potential of the ICL approach, we compared the top-performing model in the ICL approach with that of the supervised fine-tuning approach, which is widely regarded as the standard method for text classification [30,85]. The leading model in the supervised fine-tuning approach, ROBERTA, achieved a mean *F*_1_-score of 0.931 (95% CI 0.924-0.938). In comparison, the best-performing ICL model, LLAMA-3, attained a mean *F*_1_-score of 0.901 (95% CI 0.895-0.907) with only 16 annotations per class. While the *F*_1_-score difference of 0.030 corresponds to a 3.2% advantage for the fully supervised fine-tuning approach, it is worth noting that ROBERTA was developed using a training dataset of 3543 annotated sentences and a validation dataset of 500 annotated sentences, whereas LLAMA-3 used a training dataset of 32 annotated sentences and a validation dataset of 30 annotated sentences. This comparison highlighted the remarkable efficiency of the ICL approach.

As part of our analysis, we examined the fairness dimension of the models of few-shot ICL and the supervised fine-tuning approach. The results indicate that models using the supervised fine-tuning approach exhibited greater bias compared with ICL models. Specifically, with respect to TPR, supervised fine-tuning models demonstrated mean TPR disparities of 0.037, 0.077, and 0.046 across sex-, age-, and race-differentiated groups, respectively, suggesting that the TPR could vary by up to 7.7% for certain demographic subgroups. Similarly, with respect to the FPR, supervised fine-tuning models exhibited mean disparities of 0.026, 0.027, and 0.013 for sex-, age-, and race-differentiated groups, respectively. These findings indicate that supervised fine-tuning models are more likely to assign false positive classifications at uneven rates across demographic subgroups, potentially leading to the overflagging of stigmatizing language in certain populations. In contrast, ICL models tested in zero-shot, 4-shot, 8-shot, and 16-shot settings demonstrated a more balanced performance across subgroups, with no mean TPR disparities exceeding 0.016. Previous studies have shown that the supervised fine-tuning approach is susceptible to bias due to its dependence on training datasets that may have an imbalanced demographic distribution [86-88]. The primary objective of this approach is to optimize overall model performance, which can result in an emphasis on the majority or well-represented groups within the dataset. Consequently, when certain groups are underrepresented in the training data, the model’s performance tends to decline for those groups, resulting in disparities. In contrast, the ICL approach generates predictions based on the specific context presented in the prompt, minimizing the risk of inheriting and amplifying biases present in the training dataset. Therefore, the adoption of the ICL approach could contribute to more equitable detection of stigmatizing language across all patient populations.

### Clinical Adoption and Privacy Considerations

The integration of a stigmatization detection model into clinical documentation systems has the potential to enhance clinicians’ awareness of implicit biases, leading to more neutral and patient-centered documentation. However, its deployment requires careful ethical consideration. If clinicians perceive that their language is being excessively monitored or that following language guidelines imposes a cognitive burden, they may become resistant to the model or attempt to circumvent its use by omitting clinically relevant details to avoid triggering the model’s flagging system. Such reluctance could limit the model’s effectiveness in fostering more inclusive documentation practices and impact the accuracy and completeness of medical records. Therefore, it is essential to clearly communicate the model’s purpose, emphasizing its role as a supportive, educational tool rather than a punitive mechanism [89]. In addition, integrating a recommendation system that offers context-sensitive, neutral language recommendations as alternatives to flagged terms may improve clinician acceptance and facilitate smoother adoption into clinical workflows. Furthermore, real-world datasets often exhibit imbalances concerning protected attributes [87], which may result in the model disproportionately flagging certain patient groups (eg, racial minority groups and individuals with specific medical conditions) while underdetecting stigmatization in others. If left unaddressed, this could inadvertently reinforce stereotypes and exacerbate disparities in clinical documentation [90]. To mitigate these risks, the model should undergo rigorous fairness evaluations across diverse demographic groups and incorporate appropriate bias mitigation strategies before deployment [86,88]. Furthermore, we were unable to evaluate proprietary models, such as GPT-4, due to data-sharing restrictions imposed by the MIMIC-IV dataset [45]. While our findings highlight the promise of open-source LLMs in data-scarce settings, the exclusion of proprietary models limits the generalizability of our findings. Proprietary models often incorporate more advanced architectures and are trained on larger, more diverse corpora, which may allow them to outperform open-source alternatives [81]. Future studies leveraging synthetic data or institutional data-sharing agreements may be needed to assess if these proprietary models outperform open-source models in detecting stigmatizing language.

With the growing adoption of AI scribes in clinical settings, important considerations arise regarding the potential introduction or perpetuation of stigmatizing language in automatically generated clinical notes. Current AI scribing tools often operate as stand-alone systems, transcribing clinician-patient conversations and generating documentation with limited real-time oversight [91,92]. While many advanced LLMs are trained using debiasing techniques to reduce the risk of overtly harmful or abusive type of stigmatizing language (ie, junkie) [93], these methods are not explicitly designed to detect subtler forms of stigmatization commonly found in clinical narratives. The detection tool developed in this study can address this challenge in 2 ways. First, it can be applied during model development to identify and remove implicit forms of stigmatizing content from training data, thereby preventing LLMs from learning and reproducing such language. Second, it can serve as a postgeneration filter to flag potentially stigmatizing content in AI-generated clinical notes. In addition, our models can be used to design a human-AI collaborative scribing tool where AI-generated drafts are reviewed and finalized by clinicians [94]. In this context, the tool developed in this study can assist by alerting clinicians to potentially stigmatizing language, thereby promoting more neutral and patient-centered documentation. Future work should focus on validating the tool’s effectiveness in real-world clinical scribing environments and examining clinician acceptance and workflow integration.

Beyond ethical and deployment considerations, the variability of stigmatizing language across documentation types and clinical settings presents a practical consideration for deployment. Previous studies indicate that stigmatizing language is more frequently observed in history and physical notes, consultation notes, and discharge summaries compared to other types of clinical documentation and tends to be more common in relation to conditions such as substance use disorders, mental health conditions, diabetes, and obesity [8,10]. These patterns underscore the importance of accounting for documentation context and clinical setting when developing stigma detection tools. For instance, the ICL approach could be optimized by incorporating examples drawn from specific note types or clinical domains where stigmatizing language is more prevalent. Such tailoring may improve the model’s sensitivity to context-dependent expressions of bias. Moreover, recognizing that different specialties and note types reflect distinct clinician workflows, linguistic norms, and patient populations, it is essential to validate model performance across these subdomains. These validation efforts are critical to ensuring equitable performance across documentation formats and minimizing unintended disparities during deployment.

In addition to detecting stigmatizing language, LLMs can serve as valuable preprocessing tools for identifying linguistic patterns that may be integrated into downstream statistical models to explore potential associations between language use and care quality or operational decisions [15]. Furthermore, LLMs could provide real-time alerts about the potential impact of specific terms in clinical notes. These systems can draw on findings from previous research on the effects of specific terminology in clinical communication to prompt clinicians to reflect on their language choices. For example, if a term such as “aggressive” is used in a clinical note and identified by LLM as stigmatizing based on its contextual use, the system could generate a prompt indicating that the term may lead to patient discomfort or feelings of alienation. Such feedback could serve as a reflective checkpoint, prompting clinicians to reconsider their word choices and adopt more neutral, patient-centered language.

From the perspective of patient privacy and confidentiality, while deidentification is a crucial step in protecting patient privacy [46,95], the use of potentially sensitive data, such as stigmatizing language, still presents confidentiality concerns. Specifically, there are 2 key privacy risks associated with deploying machine learning–based stigmatization detection models in clinical settings. First, despite deidentification, the model may learn associations between specific linguistic patterns and particular patient populations [96]. For example, if the model systematically classifies clinical notes from a specific hospital, demographic group, or medical condition as stigmatizing, it could lead to unintended disclosures about those populations. If this information were to be combined with external data sources, there is a potential risk of reidentifying patient groups or even individuals. To mitigate such risks, implementing strict access control measures is necessary to ensure that only authorized clinicians and relevant personnel can interact with the model [96,97]. Second, the dataset used to train the model was curated based on sentences containing potentially stigmatizing words or expressions, introducing additional privacy concerns. Improper handling of the dataset during preprocessing, storage, or transfer could pose privacy risks [98]. To address this, robust data security measures should be implemented, including secure storage environments with access logging and monitoring, as well as strict protocols for data transfer to prevent unauthorized access. These safeguards are essential to balancing the benefits of stigmatization detection with the need to protect patient confidentiality in real-world clinical practice.

### Comparison With Previous Work

LLMs are increasingly being used in health care for a wide range of applications, demonstrating their capacity to process and interpret complex clinical textual data [36,74,99]. These applications encompass medical question answering, where LLMs assist clinicians and patients by providing answers to health-related queries [99], and medical text summarization, which simplifies lengthy clinical documents into concise summaries for improved decision-making [99]. In addition, LLMs are used for biomedical evidence extraction, enabling the identification of relevant research findings and coreference resolution, ensuring clarity and coherence in clinical narratives [32]. Other notable applications include medication status tracking, which monitors patient prescriptions and attribute extraction, which identifies key patient or clinical characteristics from unstructured text to support data-driven insights in health care workflows [32]. Furthermore, LLMs have been used to support clinicians in developing diagnostic reasoning, with research demonstrating that, when paired with appropriate prompting strategies, LLMs can effectively mimic common clinical reasoning processes without compromising diagnostic accuracy [100]. While these applications underscore the versatility of LLMs in advancing clinical workflow efficiency and decision-making, our manuscript addresses a distinct and underexplored challenge: detecting stigmatizing language in EHRs. Stigmatizing language within clinical documentation can perpetuate biases, undermine patient trust, and negatively impact the quality of care provided. By focusing on this critical issue, our work aims to promote bias-aware clinical documentation practices and foster equity in health care communication, addressing a significant gap in the field of medical informatics.

Sentiment analysis, a well-established area of research in NLP, has informed a wide range of clinical applications, including early studies on patient sentiment and health care provider communication patterns [101]. It shares both conceptual and methodological similarities with the task of detecting stigmatizing language. From a conceptual standpoint, sentiment analysis aims to classify the emotional tone of language, typically as positive, negative, or neutral. In contrast, stigmatization detection seeks to identify socially harmful or biased expressions. While the 2 tasks differ in their end goals, it is plausible that stigmatizing language often co-occurs with negative sentiment. Investigating whether sentiment can reliably predict stigmatization represents a promising direction for future research. However, as our dataset does not include sentiment labels, we were unable to empirically examine the potential overlap between negative sentiment and stigmatizing content. From the perspective of methodological similarity, both tasks are commonly addressed using text classification models. Indeed, the BERT-based models used in our study are widely used in sentiment analysis [102,103]. The primary methodological distinction lies in the labeling: sentiment analysis requires annotations for emotional tone, while stigmatization detection relies on annotations indicating the presence of stigmatizing content in text.

Extensive literature has documented the adverse effects of stigmatizing language in EHRs on the quality of patient care [7,8,16,20,22,24-26]. Despite this, the use of machine learning techniques to detect such language has received relatively limited attention. Sun et al [15] were among the first to apply machine learning in this context, developing a logistic regression classifier in a supervised fashion that achieved an *F*_1_-score of 0.935 on a proprietary dataset. However, their study focused primarily on the unequal use of stigmatizing language across different demographic groups, placing less emphasis on the development and optimization of the machine learning model itself. Harrigian et al [27] conducted a more technical analysis using 2 EHR datasets, exploring the application of logistic regression and BERT models for detecting stigmatizing language. Barcelona et al [28] developed and evaluated decision trees, random forests, and support vector machines for detecting stigmatizing language in labor and clinical notes.

In contrast, our research focused on evaluating and comparing the effectiveness of various zero-shot and few-shot approaches, which can operate without the need for extensive annotated data. Our findings revealed that the ICL approach significantly outperformed traditional zero-shot and few-shot text classification approaches, such as textual entailment and SetFit, while only slightly underperforming compared to the fully supervised fine-tuning approach, despite using over 100 times fewer annotated data points for training. By leveraging ICL, the reliance on large labeled datasets can be significantly diminished, enabling the development of more efficient and flexible solutions for detecting stigmatizing language across diverse clinical settings. Furthermore, our study is the first to rigorously assess the fairness dimension of machine learning classifiers specifically for stigmatizing language detection. In comparing the ICL approach to the supervised fine-tuning approach, we found that the latter is more prone to demonstrate unequal performance across specific demographic groups. However, our study did not explicitly examine methods that can be used to improve the fairness of models developed with a supervised fine-tuning approach, such as diversifying training datasets [104,105] and incorporating fairness constraints [86].

### Limitations

This study is subject to several limitations that should be acknowledged. First, the analysis was conducted using a single dataset obtained from the emergency department of the Beth Israel Deaconess Medical Center in Boston, Massachusetts. Consequently, the findings are inherently constrained by the specific characteristics of this dataset, including its geographical setting, imbalanced patient demographics, and institutional practices. Therefore, the generalizability of the models’ performance to other stigmatizing language datasets remains uncertain. Future research should aim to assess the ICL approach using larger and more diverse datasets from multiple institutions.

Second, the dataset used in this study was curated by selecting sentences that contained preidentified keywords. Stigmatizing language can manifest both explicitly through overtly negative terms and implicitly through subtle variations in tone, phrasing, or contextual framing. While our approach may excel in the detection of explicit forms of stigmatizing language, it does not fully capture more implicit forms of stigmatization. As such, our evaluation may be limited in its ability to capture the full spectrum of stigmatizing language. We hypothesize that the ICL approach has the potential to detect subtler forms of stigma by leveraging contextual information through carefully crafted prompts, as partially demonstrated in this study. However, due to the nature of the available dataset, we were unable to empirically assess the model’s generalizability to more implicit expressions of stigma. Future work should focus on developing datasets that capture a broader range of stigmatizing language. This would support more comprehensive evaluation and guide the improvement of models capable of identifying nuanced and context-dependent stigma. Moreover, the stigma detection guided prompt was intentionally designed to focus on a predefined keyword. While this strategy leverages important cues for detecting explicit forms of stigmatization, it may restrict the ICL models’ ability to capture broader contextual cues or detect more implicit forms of stigma. Future studies should develop models capable of identifying more subtle forms of stigmatization without reliance on preidentified terms.

Third, our current binary labeling framework does not explicitly address 2 important nuances. It does not explicitly consider ambiguous cases where the interpretation of stigmatization is context-dependent. The current dataset employs binary labels, stigmatizing or nonstigmatizing, without accounting for sentences that may require additional context for accurate classification. Future research could address this issue by introducing an additional classification category, such as “uncertain/requires further context,” similar to the CheXpert dataset [106]. This would enable models to flag ambiguous cases for human review or request additional contextual information to support model predictions. In addition, while collapsing distinct categories of stigmatizing language into a binary label (stigmatizing vs nonstigmatizing) enabled broader applicability and methodological comprehensiveness in this study, it may have obscured important nuances related to the impacts of different types of stigma. Previous literature has highlighted the clinical consequences of stigmatizing language [17,18], but the differential effects of specific stigma dimensions remain underexplored. In future work, we intend to systematically investigate whether specific types of stigma are differentially associated with key clinical outcomes.

Fourth, we did not examine the effectiveness of fine-tuning decoder-based models, such as LLAMA, due to the limited availability of annotated data, and as a result, their efficacy in detecting stigmatizing language remains underexplored. Future research should investigate whether fine-tuning decoder-based models can enhance performance in settings where large-scale annotated datasets are available.

Fifth, this study focused on fairness evaluation based on race, sex, and age, but did not incorporate other sociodemographic factors, such as socioeconomic status, education level, or geographic location, due to data limitations. Future research should consider a more comprehensive fairness analysis that encompasses these attributes to ensure equitable model performance across diverse populations. Sixth, while this study includes a fairness evaluation of different models for stigmatizing language detection, it does not examine the effectiveness of various bias mitigation techniques, as such an analysis would require a separate, dedicated investigation. Future research is encouraged to explore bias reduction strategies, such as diversifying training datasets [105] and implementing fairness-aware machine learning techniques [107].

Finally, we hypothesize that machine learning solutions for detecting stigmatizing language will be used as decision-support systems within clinical workflows rather than as decision-making systems due to the necessity of human oversight and the importance of ensuring that narratives within EHRs are validated by clinicians. While this study discusses several potential ethical implications for the clinical deployment of the stigmatization detection model, it did not address several critical factors essential for effective human-AI collaboration in this context, such as varying levels of automation, system usability, and the potential for automation bias. Future research should explore these factors to ensure the successful integration of such systems into clinical workflows, thereby optimizing decision-making processes and enhancing the efficiency and effectiveness of stigmatizing language detection.

### Conclusions

We explored the potential of the ICL approach for detecting stigmatizing language in EHRs, particularly in scenarios where annotated data is limited. Our findings demonstrate that ICL approaches perform significantly better than traditional zero-shot and few-shot methods, such as textual entailment and SetFit. Moreover, our results underscore the critical importance of prompt engineering within the ICL approach. The novel prompting strategy, the stigma detection guided prompt, developed in this study, significantly enhances the detection of stigmatizing language by incorporating common linguistic characteristics of such language and guiding the model’s attention to specific keywords. When compared with the supervised fine-tuning approach, ICL has demonstrated competitive performance as well as superior fairness metrics, with lower performance disparities across different demographic groups. In summary, ICL is emerging as a robust and flexible solution for detecting stigmatizing language in EHRs, offering a more efficient, effective, and equitable alternative than conventional machine learning approaches.

## Appendix

Multimedia Appendix 1 Dataset description and stigmatizing language examples.

Multimedia Appendix 2 Detailed code implementation for the prompting strategies.

Multimedia Appendix 3 Paired *t* test comparison of top-performing models in zero-shot, few-shot, and supervised fine-tuning approaches.

Multimedia Appendix 4 Performance metrics of the few-shot in-context learning models.

Multimedia Appendix 5 Performance and performance disparities of models across sex, age, and race demographic attributes.

## Abbreviations

AI: artificial intelligence
AUC-ROC: area under the receiver operating characteristic curve
BERT: Bidirectional Encoder Representations from Transformers
CARP: clue and reasoning prompting
COT: chain of thought
EHR: electronic health record
FPR: false positive rate
ICL: in-context learning
LLM: large language model
MiMIC: Medical Information Mart for Intensive Care
NLP: natural language processing
TPR: true positive rate

## Data Availability

The datasets analyzed during this study are available in the PhysioNet repository [27]. The code supporting this study is intended for public release via the PhysioNet repository in the future. In the meantime, access can be requested directly from the corresponding author.
