# Supplementary material for: Efficient Detection of Stigmatizing Language in Electronic Health Records via In-Context Learning: Comparative Analysis and Validation Study
Source: JMIR Med Inform. 2025 Aug 18;13:e68955. doi: 10.2196/68955 (PMC12402740; doi:10.2196/68955)
Supplement: Multimedia Appendix 1 [file medinform_v13i1e68955_app1.docx]

## Multimedia Appendix-1: Dataset Description and Stigmatizing Language Examples.

The dataset utilized in this study was originally curated by Harrigian et al. [1]. Harrigian et al. originally categorized 5,043 sentences from the discharge summaries in the MIMIC-IV database and framed stigmatizing language detection as three independent classification tasks, each targeting different types of stigmatizing language. These tasks included: ‘credibility and obstinacy’ (with labels Disbelief, Difficult, Exclude), ‘compliance’ (with labels Negative, Neutral, Positive), and ‘descriptors’ (with labels Negative, Neutral, Positive, Exclude). Table 1 below presents the original classification taxonomy and associated examples as developed by Harrigian et al. [1]. The taxonomy is developed by the study team of Harrigian et al, drawing upon previous literature [2,3].

In our study, these dimensions were collapsed into a binary classification by treating the labels “Disbelief”, by treating the labels “Disbelief”, “Difficult”, and “Negative” as stigmatizing, and the remaining labels as non-stigmatizing. From a conceptual perspective, we recognize that different dimensions of stigma represent important nuances, however, there is currently no universally accepted taxonomy for categorizing stigmatizing language [1,3,4]. To ensure the broad applicability of our study, we opted for a conventional binary classification framework consistent with prior work [3,5,6]. This alignment also enhances the comparability and interpretability of results across studies. In addition, a binary formulation enables more consistent and efficient model training, allowing for robust comparisons across a broad range of models across zero-shot, few-shot, and supervised learning.

**Table 1.** Classification taxonomy of stigmatizing language and associated examples, originally developed by Harrigian et al. [1].

| **Stigma Type** | **Class** | **Definition** | **Examples** |
| --- | --- | --- | --- |
| Credibility & Obstinacy | Disbelief | Insinuates doubt about a patient’s stated testimony. | adamant he doesn’t smoke; claims to see a therapist |
|  | Difficult | Describes patient (or patient’s family) perspective as inflexible/difficult/entrenched, typically with respect to their intentions. | insists on being admitted; adamantly opposed to limiting fruit intake |
|  | Exclude | Word/phrase is not used to characterize the patient or describe the patient’s behavior; may refer to medical condition or treatment or to another person or context. | patient’s friend insisted she go to the hospital; test claims submitted to insurance |
| Compliance | Negative | Patient not, unlikely to, or questionably following medical advice. | adherence to therapeutic medication is unclear; mother declines vaccines; struggles with medication and follow-up compliance |
|  | Neutral | Not used to describe whether the patient is not following medical advice or rejecting treatment; often used to describe generically some future plan involving a hypothetical. Alternatively, see Exclude (Credibility & Obstinacy). | discussed the medication compliance; school refuses to provide adequate accommodations; feels that her parents’ health has declined |
|  | Positive | Patient following medical advice. | continues to be compliant with aspirin regimen; reports excellent adherence |
| Descriptors | Negative | Patient’s demeanor or behavior is cast in a negative light; insinuates the patient is not being forthright or transparent; patient may be falsifying symptoms to get something they want. | drug-seeking behavior; concern for secondary gain; unwilling to meet with case manager; unfortunately a poor historian |
|  | Neutral | Negation of negative descriptors; insinuates the patient was expected to have a negative demeanor or be difficult to interact with. | his mother is the primary historian; interactive and cooperative; not combative or belligerent; dad seems angry with patient at times |
|  | Positive | Patient’s demeanor or behavior is described in a positive light; patient is easy to interact with. | lovely 80 year old woman; well-groomed and holds good eye contact; pleasant and appropriate interaction with staff |
|  | Exclude | Patient self-description or description of another individual. Alternatively, see Exclude (Credibility & Obstinacy). | does not want providers to think she’s malingering; reports feeling angry before her period; lives on pleasant avenue downtown |

References

1. Harrigian K, Zirikly A, Chee B, Ahmad A, Links A, Saha S, Beach MC, Dredze M. Characterization of Stigmatizing Language in Medical Records. In: Rogers A, Boyd-Graber J, Okazaki N, editors. Proceedings of the 61st Annual Meeting of the Association for Computational Linguistics (Volume 2: Short Papers) Toronto, Canada: Association for Computational Linguistics; 2023. p. 312–329. doi: 10.18653/v1/2023.acl-short.28

2. Beach MC, Saha S, Park J, Taylor J, Drew P, Plank E, Cooper LA, Chee B. Testimonial Injustice: Linguistic Bias in the Medical Records of Black Patients and Women. J Gen Intern Med 2021 Jun;36(6):1708–1714. PMID:33754318

3. Sun M, Oliwa T, Peek ME, Tung EL. Negative Patient Descriptors: Documenting Racial Bias In The Electronic Health Record. Health Affairs Health Affairs; 2022 Feb;41(2):203–211. doi: 10.1377/hlthaff.2021.01423

4. Park J, Saha S, Chee B, Taylor J, Beach MC. Physician Use of Stigmatizing Language in Patient Medical Records. JAMA Network Open 2021 Jul 14;4(7):e2117052. doi: 10.1001/jamanetworkopen.2021.17052

5. Kelly JF, Westerhoff CM. Does it matter how we refer to individuals with substance-related conditions? A randomized study of two commonly used terms. Int J Drug Policy 2010 May;21(3):202–207. PMID:20005692

6. P Goddu A, O’Conor KJ, Lanzkron S, Saheed MO, Saha S, Peek ME, Haywood C, Beach MC. Do Words Matter? Stigmatizing Language and the Transmission of Bias in the Medical Record. J Gen Intern Med 2018 May;33(5):685–691. PMID:29374357
