# Supplementary material for: Efficient Detection of Stigmatizing Language in Electronic Health Records via In-Context Learning: Comparative Analysis and Validation Study
Source: JMIR Med Inform. 2025 Aug 18;13:e68955. doi: 10.2196/68955 (PMC12402740; doi:10.2196/68955)
Supplement: Multimedia Appendix 2 [file medinform_v13i1e68955_app2.docx]

## Multimedia Appendix-2: Detailed Code Implementation for the Prompting Strategies.

Table 1. The code implementation for the prompting strategies.

| **Prompting Strategy** | **Code for Prompting Template** |
| --- | --- |
| Generic | Template= [  {"role": "system", "content": "You are an expert in detecting stigmatizing language."},  {"role": "user", "content": f""" Input: "{statement}". Choose your answer: Based on the above sentence, does the text convey stigmatization? Yes/No """}] |
| COT | Template=[  {"role": "system", "content": "You are an expert in detecting stigmatizing language."},  {"role": "user", "content": f""" Input: ‘{statement}’ Determine if the input contains stigmatizing language. Let’s think step by step. Reasoning: ‘{reasoning}.’ Therefore, the answer (yes or no) is:"""}] |
| CARP | Template=[  {"role": "system", "content": "You are an expert in detecting stigmatizing language."},  {"role": "user", "content": f"""  First, list clues (e.g., keywords, phrases, contextual information, semantic relations, tones, references) that support the determination of stigmatization in the input.  Second, deduce the diagnostic reasoning process from the premises (i.e., clues and input) that support the stigmatization determination.  Third, based on the clues, reasoning, and input, determine whether the input conveys stigmatization.  Input: ‘{statement}’  Answer with 'yes' or 'no’.  Answer:""""}] |
| Stigma Detection Guided Prompt | Template=[  {"role": "system", "content": "You are an expert in detecting stigmatizing language."},  {"role": "user", "content": f""" Stigmatizing language can exhibit the following characteristics:  1) Questioning credibility: Implication of physician disbelief in patient reports of their own experiences or behaviors.  2) Disapproval: Highlights poor reasoning, decision-making, or self-care, often in a way that suggests the patient is unreasonable.  3) Stereotyping: Quoting incorrect grammar or unsophisticated terms.  4) Difficult patient: Inclusion of details with questionable clinical significance that portray the patient as belligerent or otherwise suggest that the physician is annoyed.  5) Unilateral decisions: Language that emphasizes physician authority.  Input: ‘{statement}’  Keyword: ‘{keyword}.’  Does the keyword in the input convey stigmatization? Answer with 'Yes' or 'No'.  Answer:"  """} |
