# Supplementary material for: Efficient Detection of Stigmatizing Language in Electronic Health Records via In-Context Learning: Comparative Analysis and Validation Study
Source: JMIR Med Inform. 2025 Aug 18;13:e68955. doi: 10.2196/68955 (PMC12402740; doi:10.2196/68955)
Supplement: Multimedia Appendix 3 [file medinform_v13i1e68955_app3.docx]

## Multimedia Appendix-3: Paired t-test Comparison of Top-performing Models in Zero-Shot, Few-Shot, and Supervised Fine-Tuning Approaches.

The paired t-test results for comparing top-performing models in zero-shot and few-shot settings, as well as the comparison between the best-performing model in the ICL approach and the best-performing supervised model, are presented in Tables 1, 2, and 3, respectively.

Table 1. Paired t-test results for comparing top-performing models in textual entailment and zero-shot ICL.

| **Comparison** | **Metric** | **t-statistic** | **Sample size** | **Mean difference** | **P-value** |
| --- | --- | --- | --- | --- | --- |
| Deberta-M vs. GEMMA-2 coupled with Stigma Detection Guided Prompt | F1 | -74.186 | 10 | -0.136 | <.001 |
|  | Precision | -50.286 | 10 | -0.125 | <.001 |
|  | Recall | -70.761 | 10 | -0.146 | <.001 |

Table 2. Paired t-test results for comparing top-performing models in SetFit and few-shot ICL.

| **Number of annotations per label** | **Comparison** | **Metric** | **t-statistic** | **Sample size** | **Mean difference** | **P-value** |
| --- | --- | --- | --- | --- | --- | --- |
| 4 | MINILM-V2 vs. LLAMA 3 coupled with Stigma Detection Guided Prompt | F1 | -40.651 | 10 | -0.155 | <.001 |
|  |  | Precision | -40.338 | 10 | -0.138 | <.001 |
|  |  | Recall | -61.25 | 10 | -0.165 | <.001 |
| 8 | E5-V2 vs. LLAMA 3 coupled with Stigma Detection Guided Prompt | F1 | -60.140 | 10 | -0.158 | <.001 |
|  |  | Precision | -45.95 | 10 | -0.124 | <.001 |
|  |  | Recall | -59.267 | 10 | -0.192 | <.001 |
| 16 | E5-V2 vs. LLAMA 3 coupled with Stigma Detection Guided Prompt | F1 | -44.614 | 10 | -0.101 | <.001 |
|  |  | Precision | -17.096 | 10 | -0.052 | <.001 |
|  |  | Recall | -80.413 | 10 | -0.152 | <.001 |

Table 3. Paired t-test results for comparing top-performing models in supervised fine-tuning and few-shot ICL.

| **Comparison** | **Metric** | **t-statistic** | **Sample size** | **Mean difference** | **P-value** |
| --- | --- | --- | --- | --- | --- |
| ROBERTA vs. LAMMA-3 coupled with Stigma Detection Guided Prompt (16 annotated data per class) | F1 | 11.03 | 10 | 0.029 | <.001 |
|  | Precision | 26.325 | 10 | 0.086 | <.001 |
|  | Recall | -9.695 | 10 | -0.030 | <.001 |
