# Supplementary material for: Efficient Detection of Stigmatizing Language in Electronic Health Records via In-Context Learning: Comparative Analysis and Validation Study
Source: JMIR Med Inform. 2025 Aug 18;13:e68955. doi: 10.2196/68955 (PMC12402740; doi:10.2196/68955)
Supplement: Multimedia Appendix 4 [file medinform_v13i1e68955_app4.docx]

## Multimedia Appendix-4: Performance Metrics of the Few-shot ICL Models.

Table 1. Performance metrics of the few-shot ICL models using Generic prompting strategy.

| **Prompting Strategy** | **4 annotated data per class** | | | | |
| --- | --- | --- | --- | --- | --- |
| **Generic** | **Model** | **Accuracy** | **F1** | **Precision** | **Recall** |
|  | LLAMA-3 | 0.748±0.008 | 0.789±0.006 | 0.855±0.007 | 0.733±0.008 |
|  | FLAN-T5 | 0.693±0.005 | 0.691±0.007 | 0.712±0.005 | 0.672±0.008 |
|  | GEMMA-2 | 0.711±0.012 | 0.719±0.007 | 0.731±0.008 | 0.708±0.006 |
|  | MISTRAL-0.2 | 0.725±0.007 | 0.730±0.006 | 0.752±0.005 | 0.710±0.004 |
|  | BIO-LLAMA-3 | 0.766±0.009 | 0.820±0.008 | 0.832±0.011 | 0.809±0.008 |
|  | **8 annotated data per class** | | | | |
|  | **Model** | **Accuracy** | **F1** | **Precision** | **Recall** |
|  | LLAMA-3 | 0.792±0.009 | 0.832±0.008 | 0.865±0.010 | 0.802±0.012 |
|  | FLAN-T5 | 0.707±0.008 | 0.701±0.008 | 0.712±0.009 | 0.691±0.009 |
|  | GEMMA-2 | 0.721±0.008 | 0.739±0.009 | 0.757±0.011 | 0.723±0.007 |
|  | MISTRAL-0.2 | 0.705±0.009 | 0.720±0.010 | 0.731±0.013 | 0.709±0.012 |
|  | BIO-LLAMA-3 | 0.773±0.005 | 0.826±0.004 | 0.863±0.010 | 0.792±0.007 |
|  | **16 annotated data per class** | | | | |
|  | **Model** | **Accuracy** | **F1** | **Precision** | **Recall** |
|  | LLAMA-3 | 0.816±0.008 | 0.855±0.006 | 0.869±0.009 | 0.842±0.012 |
|  | FLAN-T5 | 0.751±0.005 | 0.762±0.006 | 0.775±0.004 | 0.749±0.007 |
|  | GEMMA-2 | 0.762±0.009 | 0.773±0.008 | 0.765±0.005 | 0.782±0.008 |
|  | MISTRAL-0.2 | 0.801±0.010 | 0.822±0.007 | 0.832±0.008 | 0.813±0.004 |
|  | BIO-LLAMA-3 | 0.790±0.006 | 0.851±0.006 | 0.901±0.011 | 0.807±0.004 |

Table 2. Performance metrics of the few-shot ICL models using COT prompting strategy.

| **Prompting Strategy** | **4 annotated data per class** | | | | |
| --- | --- | --- | --- | --- | --- |
| **COT** | **Model** | **Accuracy** | **F1** | **Precision** | **Recall** |
|  | LLAMA-3 | 0.725±0.008 | 0.756±0.004 | 0.809±0.007 | 0.709±0.004 |
|  | FLAN-T5 | - | - | - | - |
|  | GEMMA-2 | 0.751±0.004 | 0.796±0.006 | 0.810±0.005 | 0.783±0.005 |
|  | MISTRAL-0.2 | 0.713±0.003 | 0.742±0.005 | 0.753±0.006 | 0.730±0.006 |
|  | BIO-LLAMA-3 | 0.716±0.007 | 0.743±0.005 | 0.782±0.005 | 0.707±0.007 |
|  | **8 annotated data per class** | | | | |
|  | **Model** | **Accuracy** | **F1** | **Precision** | **Recall** |
|  | LLAMA-3 | 0.831±0.009 | 0.823±0.007 | 0.827±0.010 | 0.819±0.012 |
|  | FLAN-T5 | - | - | - | - |
|  | GEMMA-2 | 0.795±0.008 | 0.826±0.005 | 0.843±0.007 | 0.811±0.005 |
|  | MISTRAL-0.2 | 0.751±0.006 | 0.767±0.007 | 0.773±0.004 | 0.762±.008 |
|  | BIO-LLAMA-3 | 0.814±0.008 | 0.835±0.006 | 0.840±0.008 | 0.831±0.005 |
|  | **16 annotated data per class** | | | | |
|  | **Model** | **Accuracy** | **F1** | **Precision** | **Recall** |
|  | LLAMA-3 | - | - | - | - |
|  | FLAN-T5 | - | - | - | - |
|  | GEMMA-2 | - | - | - | - |
|  | MISTRAL-0.2 | 0.769±0.008 | 0.781±0.006 | 0.794±0.009 | 0.771±0.004 |
|  | BIO-LLAMA-3 | - | - | - | - |

Table 3. Performance metrics of the few-shot ICL models using CARP prompting strategy.

| **Prompting Strategy** | **4 annotated data per class** | | | | |
| --- | --- | --- | --- | --- | --- |
| **CARP** | **Model** | **Accuracy** | **F1** | **Precision** | **Recall** |
|  | LLAMA-3 | 0.804±0.008 | 0.837±0.005 | 0.853±0.009 | 0.822±0.004 |
|  | FLAN-T5 | - | - | - | - |
|  | GEMMA-2 | 0.754±0.007 | 0.795±0.006 | 0.804±0.005 | 0.786±0.006 |
|  | MISTRAL-0.2 | 0.718±0.005 | 0.728±0.008 | 0.734±0.006 | 0.722±0.005 |
|  | BIO-LLAMA-3 | 0.772±0.007 | 0.813±0.009 | 0.842±0.007 | 0.786±0.008 |
|  | **8 annotated data per class** | | | | |
|  | **Model** | **Accuracy** | **F1** | **Precision** | **Recall** |
|  | LLAMA-3 | 0.851±0.005 | 0.876±0.004 | 0.884±0.006 | 0.869±0.003 |
|  | FLAN-T5 | - | - | - | - |
|  | GEMMA-2 | 0.796±0.004 | 0.825±0.009 | 0.834±0.008 | 0.815±0.006 |
|  | MISTRAL-0.2 | 0.722±0.005 | 0.747±0.006 | 0.757±0.005 | 0.738±0.007 |
|  | BIO-LLAMA-3 | 0.816±0.006 | 0.849±0.008 | 0.867±0.005 | 0.832±0.006 |
|  | **16 annotated data per class** | | | | |
|  | **Model** | **Accuracy** | **F1** | **Precision** | **Recall** |
|  | LLAMA-3 | - | - | - | - |
|  | FLAN-T5 | - | - | - | - |
|  | GEMMA-2 | - | - | - | - |
|  | MISTRAL-0.2 | 0.712±0.006 | 0.731±0.005 | 0.743±0.004 | 0.720±0.005 |
|  | BIO-LLAMA-3 | - | - | - | - |

Table 4. Performance metrics of the few-shot ICL models using Stigma Detection Guided Prompt prompting strategy.

| **Prompting Strategy** | **4 annotated data per class** | | | | |
| --- | --- | --- | --- | --- | --- |
| **Stigma Detection Guided Prompt** | **Model** | **Accuracy** | **F1** | **Precision** | **Recall** |
|  | LLAMA-3 | 0.843±0.006 | 0.874±0.010 | 0.854±0.009 | 0.889±0.005 |
|  | FLAN-T5 | 0.742±0.004 | 0.782±0.006 | 0.771±0.005 | 0.794±0.005 |
|  | GEMMA-2 | 0.843±0.009 | 0.872±0.007 | 0.862±0.007 | 0.883±0.005 |
|  | MISTRAL-0.2 | 0.805±0.007 | 0.849±0.005 | 0.835±0.006 | 0.864±0.007 |
|  | BIO-LLAMA-3 | 0.804±0.011 | 0.861±0.010 | 0.839±0.007 | 0.884±0.005 |
|  | **8 annotated data per class** | | | | |
|  | **Model** | **Accuracy** | **F1** | **Precision** | **Recall** |
|  | LLAMA-3 | 0.855±0.007 | 0.892±0.006 | 0.873±0.007 | 0.912±0.008 |
|  | FLAN-T5 | 0.768±0.005 | 0.805±0.008 | 0.782±0.007 | 0.829±0.005 |
|  | GEMMA-2 | 0.849±0.004 | 0.881±0.005 | 0.870±0.004 | 0.891±0.003 |
|  | MISTRAL-0.2 | 0.820±0.006 | 0.857±0.004 | 0.853±0.006 | 0.862±0.005 |
|  | BIO-LLAMA-3 | 0.815±0.008 | 0.870±0.007 | 0.848±0.009 | 0.893±0.005 |
|  | **16 annotated data per class** | | | | |
|  | **Model** | **Accuracy** | **F1** | **Precision** | **Recall** |
|  | LLAMA-3 | 0.877±0.007 | 0.901±0.006 | 0.863±0.008 | 0.943±0.004 |
|  | FLAN-T5 | 0.828±0.003 | 0.842±0.004 | 0.833±0.006 | 0.851±0.005 |
|  | GEMMA-2 | 0.861±0.005 | 0.887±0.006 | 0.876±0.006 | 0.898±0.007 |
|  | MISTRAL-0.2 | 0.867±0.007 | 0.899±0.005 | 0.871±0.004 | 0.929±0.004 |
|  | BIO-LLAMA-3 | 0.827±0.010 | 0.892±0.009 | 0.865±0.007 | 0.921±0.005 |
