# Supplementary material for: Efficient Detection of Stigmatizing Language in Electronic Health Records via In-Context Learning: Comparative Analysis and Validation Study
Source: JMIR Med Inform. 2025 Aug 18;13:e68955. doi: 10.2196/68955 (PMC12402740; doi:10.2196/68955)
Supplement: Multimedia Appendix 5 [file medinform_v13i1e68955_app5.docx]

## Multimedia Appendix-5: Performance and Performance Disparities of Models across Sex, Age, and Race Demographic Attributes.

Table 1. Performance and performance disparities for models utilizing supervised fine-tuning approach.

| **Model** | **Attributes** | **TPR** | **TPR Disparity** | **FPR** | **FPR Disparity** | **F1** | **F1 Disparity** |
| --- | --- | --- | --- | --- | --- | --- | --- |
| ROBERTA | Male | 0.951±0.007 | 0.049 | 0.142±0.002 | -0.018 | 0.946±0.006 | 0.043 |
|  | Female | 0.902±0.005 | -0.049 | 0.160±0.004 | 0.018 | 0.903±0.008 | -0.043 |
|  | 0-25 | 0.835±0.009 | -0.108 | 0.162±0.003 | 0.022 | 0.862±0.005 | -0.066 |
|  | 26-50 | 0.947±0.006 | 0.005 | 0.142±0.003 | 0.001 | 0.922±0.004 | -0.005 |
|  | 51-75 | 0.955±0.008 | 0.013 | 0.138±0.002 | -0.003 | 0.957±0.007 | 0.030 |
|  | 75 plus | 0.938±0.003 | -0.004 | 0.139±0.004 | -0.002 | 0.933±0.009 | 0.006 |
|  | White | 0.922±0.009 | 0.029 | 0.140±0.001 | -0.008 | 0.937±0.003 | 0.017 |
|  | Black | 0.891±0.007 | -0.002 | 0.148±0.003 | 0.000 | 0.920±0.003 | 0.000 |
|  | Hispanic | 0.893±0.004 | 0.000 | 0.156±0.003 | 0.008 | 0.932±0.008 | 0.012 |
|  | Asian | 0.849±0.006 | -0.044 | 0.176±0.002 | 0.028 | 0.856±0.003 | -0.064 |
|  | Other/Uknown | 0.902±0.003 | 0.009 | 0.143±0.004 | -0.005 | 0.912±0.009 | -0.008 |
| BERT | Male | 0.925±0.009 | 0.051 | 0.134±0.002 | -0.016 | 0.930±0.005 | 0.016 |
|  | Female | 0.874±0.008 | -0.051 | 0.150±0.003 | 0.016 | 0.914±0.006 | -0.016 |
|  | 0-25 | 0.807±0.007 | -0.058 | 0.160±0.001 | 0.016 | 0.851±0.004 | -0.048 |
|  | 26-50 | 0.896±0.005 | 0.032 | 0.142±0.003 | -0.002 | 0.927±0.008 | 0.028 |
|  | 51-75 | 0.904±0.006 | 0.040 | 0.138±0.004 | -0.006 | 0.940±0.003 | 0.041 |
|  | 75 plus | 0.833±0.003 | -0.032 | 0.146±0.003 | 0.002 | 0.871±0.006 | -0.028 |
|  | White | 0.895±0.004 | 0.016 | 0.150±0.005 | 0.000 | 0.928±0.009 | 0.012 |
|  | Black | 0.877±0.009 | -0.002 | 0.138±0.002 | -0.012 | 0.886±0.004 | -0.030 |
|  | Hispanic | 0.879±0.008 | 0.000 | 0.154±0.003 | 0.004 | 0.916±0.007 | 0.000 |
|  | Asian | 0.876±0.007 | -0.003 | 0.158±0.001 | 0.008 | 0.910±0.009 | -0.006 |
|  | Other/Uknown | 0.882±0.005 | 0.003 | 0.128±0.002 | -0.022 | 0.920±0.003 | 0.004 |
| BERT-BIO | Male | 0.914±0.006 | 0.022 | 0.153±0.003 | -0.035 | 0.882±0.008 | 0.003 |
|  | Female | 0.892±0.003 | -0.022 | 0.188±0.003 | 0.035 | 0.879±0.005 | -0.003 |
|  | 0-25 | 0.840±0.009 | -0.075 | 0.177±0.004 | 0.022 | 0.840±0.004 | -0.058 |
|  | 26-50 | 0.926±0.008 | 0.011 | 0.148±0.001 | -0.008 | 0.884±0.007 | -0.014 |
|  | 51-75 | 0.930±0.007 | 0.015 | 0.122±0.001 | -0.034 | 0.912±0.009 | 0.014 |
|  | 75 plus | 0.904±0.005 | -0.011 | 0.163±0.003 | 0.008 | 0.919±0.003 | 0.022 |
|  | White | 0.938±0.006 | 0.037 | 0.158±0.004 | 0.000 | 0.910±0.003 | 0.015 |
|  | Black | 0.910±0.003 | 0.009 | 0.158±0.002 | 0.000 | 0.918±0.005 | 0.023 |
|  | Hispanic | 0.877±0.005 | -0.024 | 0.181±0.004 | 0.023 | 0.864±0.006 | -0.031 |
|  | Asian | 0.901±0.004 | 0.000 | 0.164±0.005 | 0.006 | 0.885±0.008 | -0.010 |
|  | Other/Uknown | 0.898±0.009 | -0.003 | 0.131±0.001 | -0.027 | 0.895±0.003 | 0.000 |
| ROBERTA-XLM | Male | 0.934±0.007 | 0.033 | 0.164±0.002 | -0.022 | 0.907±0.005 | 0.006 |
|  | Female | 0.901±0.005 | -0.033 | 0.186±0.002 | 0.022 | 0.900±0.006 | -0.006 |
|  | 0-25 | 0.823±0.007 | -0.090 | 0.165±0.004 | 0.023 | 0.811±0.005 | -0.086 |
|  | 26-50 | 0.933±0.005 | 0.021 | 0.142±0.003 | 0.000 | 0.919±0.006 | 0.022 |
|  | 51-75 | 0.939±0.006 | 0.026 | 0.142±0.004 | 0.000 | 0.927±0.009 | 0.029 |
|  | 75 plus | 0.892±0.005 | -0.021 | 0.133±0.005 | -0.009 | 0.876±0.003 | -0.022 |
|  | White | 0.941±0.008 | 0.052 | 0.161±0.002 | 0.000 | 0.896±0.009 | 0.017 |
|  | Black | 0.910±0.007 | 0.021 | 0.144±0.001 | -0.017 | 0.904±0.005 | 0.026 |
|  | Hispanic | 0.859±0.005 | -0.030 | 0.157±0.003 | -0.004 | 0.846±0.006 | -0.032 |
|  | Asian | 0.889±0.006 | 0.000 | 0.205±0.001 | 0.044 | 0.867±0.005 | -0.011 |
|  | Other/Uknown | 0.872±0.003 | -0.017 | 0.164±0.001 | 0.003 | 0.878±0.000 | 0.000 |
| BERT-BIOMED | Male | 0.918±0.004 | 0.032 | 0.186±0.003 | -0.039 | 0.913±0.007 | 0.040 |
|  | Female | 0.886±0.009 | -0.032 | 0.225±0.004 | 0.039 | 0.873±0.006 | -0.040 |
|  | 0-25 | 0.823±0.007 | -0.053 | 0.223±0.002 | 0.038 | 0.815±0.005 | -0.053 |
|  | 26-50 | 0.917±0.005 | 0.042 | 0.180±0.001 | -0.005 | 0.898±0.006 | 0.030 |
|  | 51-75 | 0.910±0.006 | 0.035 | 0.190±0.004 | 0.005 | 0.884±0.007 | 0.016 |
|  | 75 plus | 0.841±0.003 | -0.035 | 0.155±0.003 | -0.030 | 0.852±0.006 | -0.016 |
|  | White | 0.906±0.004 | 0.010 | 0.198±0.003 | 0.004 | 0.888±0.009 | 0.011 |
|  | Black | 0.924±0.009 | 0.028 | 0.182±0.003 | -0.012 | 0.894±0.008 | 0.016 |
|  | Hispanic | 0.879±0.008 | -0.017 | 0.206±0.002 | 0.012 | 0.877±0.003 | 0.000 |
|  | Asian | 0.832±0.007 | -0.064 | 0.194±0.006 | 0.000 | 0.788±0.005 | -0.089 |
|  | Other/Unknown | 0.896±0.005 | 0.000 | 0.161±0.003 | -0.033 | 0.856±0.005 | -0.021 |

Table 2. Performance and performance disparities for models utilizing zero-shot ICL approach.

| **Model** | **Attributes** | **TPR** | **TPR**  **Disparity** | **FPR** | **FPR**  **Disparity** | **F1** | **F1**  **Disparity** |
| --- | --- | --- | --- | --- | --- | --- | --- |
| LLAMA-3 | Male | 0.805±0.004 | -0.005 | 0.192±0.004 | 0.003 | 0.803±0.003 | -0.006 |
|  | Female | 0.810±0.006 | 0.005 | 0.190±0.002 | -0.003 | 0.809±0.004 | 0.006 |
|  | 0-25 | 0.814±0.003 | -0.004 | 0.184±0.003 | 0.002 | 0.802±0.005 | -0.010 |
|  | 26-50 | 0.816±0.002 | -0.002 | 0.180±0.003 | -0.002 | 0.808±0.006 | -0.004 |
|  | 51-75 | 0.829±0.006 | 0.012 | 0.177±0.005 | -0.005 | 0.827±0.004 | 0.015 |
|  | 75 plus | 0.820±0.005 | 0.002 | 0.190±0.003 | 0.008 | 0.817±0.004 | 0.004 |
|  | White | 0.822±0.003 | 0.003 | 0.186±0.002 | -0.004 | 0.813±0.006 | -0.006 |
|  | Black | 0.819±0.005 | 0.000 | 0.192±0.005 | 0.002 | 0.820±0.003 | 0.001 |
|  | Hispanic | 0.823±0.002 | 0.003 | 0.198±0.004 | 0.008 | 0.818±0.005 | -0.002 |
|  | Asian | 0.815±0.006 | -0.005 | 0.190±0.003 | 0.000 | 0.820±0.004 | 0.001 |
|  | Other/Uknown | 0.808±0.002 | -0.012 | 0.186±0.004 | -0.004 | 0.819±0.009 | 0.000 |
| FLAN-T5 | Male | 0.748±0.004 | -0.002 | 0.246±0.006 | -0.007 | 0.742±0.005 | 0.004 |
|  | Female | 0.750±0.007 | 0.002 | 0.254±0.006 | 0.007 | 0.738±0.006 | -0.004 |
|  | 0-25 | 0.750±0.005 | 0.001 | 0.253±0.005 | 0.005 | 0.725±0.004 | -0.018 |
|  | 26-50 | 0.747±0.006 | -0.002 | 0.237±0.004 | -0.011 | 0.742±0.006 | -0.001 |
|  | 51-75 | 0.747±0.005 | -0.001 | 0.242±0.003 | -0.005 | 0.750±0.005 | 0.007 |
|  | 75 plus | 0.752±0.003 | 0.004 | 0.259±0.002 | 0.011 | 0.744±0.006 | 0.001 |
|  | White | 0.748±0.007 | -0.007 | 0.235±0.003 | -0.006 | 0.738±0.007 | -0.007 |
|  | Black | 0.756±0.007 | 0.000 | 0.241±0.005 | 0.000 | 0.756±0.003 | 0.011 |
|  | Hispanic | 0.758±0.004 | 0.002 | 0.257±0.003 | 0.016 | 0.745±0.004 | 0.000 |
|  | Asian | 0.744±0.005 | -0.012 | 0.232±0.004 | -0.010 | 0.753±0.005 | 0.008 |
|  | Other/Uknown | 0.760±0.003 | 0.004 | 0.241±0.003 | 0.000 | 0.744±0.002 | -0.001 |
| GEMMA-2 | Male | 0.780±0.004 | -0.003 | 0.201±0.002 | 0.008 | 0.774±0.004 | -0.010 |
|  | Female | 0.783±0.006 | 0.003 | 0.194±0.004 | -0.008 | 0.784±0.003 | 0.010 |
|  | 0-25 | 0.780±0.007 | 0.000 | 0.195±0.003 | 0.001 | 0.786±0.002 | 0.002 |
|  | 26-50 | 0.788±0.004 | 0.009 | 0.196±0.004 | 0.002 | 0.772±0.006 | -0.012 |
|  | 51-75 | 0.775±0.005 | -0.004 | 0.176±0.006 | -0.018 | 0.796±0.007 | 0.013 |
|  | 75 plus | 0.779±0.006 | 0.000 | 0.193±0.005 | -0.001 | 0.781±0.005 | -0.002 |
|  | White | 0.785±0.003 | 0.010 | 0.186±0.002 | -0.003 | 0.765±0.008 | -0.016 |
|  | Black | 0.787±0.006 | 0.012 | 0.188±0.003 | -0.001 | 0.781±0.005 | -0.001 |
|  | Hispanic | 0.775±0.007 | 0.000 | 0.204±0.004 | 0.015 | 0.790±0.002 | 0.008 |
|  | Asian | 0.775±0.005 | 0.000 | 0.209±0.005 | 0.019 | 0.785±0.003 | 0.004 |
|  | Other/Uknown | 0.771±0.006 | -0.005 | 0.189±0.005 | 0.000 | 0.781±0.006 | 0.000 |
| MISTRAL-0.2 | Male | 0.741±0.003 | -0.007 | 0.248±0.007 | -0.005 | 0.741±0.007 | -0.006 |
|  | Female | 0.748±0.006 | 0.007 | 0.253±0.008 | 0.005 | 0.746±0.004 | 0.006 |
|  | 0-25 | 0.731±0.002 | -0.012 | 0.236±0.003 | 0.005 | 0.731±0.005 | -0.015 |
|  | 26-50 | 0.740±0.002 | -0.003 | 0.235±0.004 | 0.004 | 0.748±0.005 | 0.003 |
|  | 51-75 | 0.755±0.004 | 0.012 | 0.215±0.006 | -0.016 | 0.748±0.006 | 0.002 |
|  | 75 plus | 0.746±0.004 | 0.003 | 0.228±0.005 | -0.004 | 0.744±0.007 | -0.002 |
|  | White | 0.755±0.005 | 0.011 | 0.238±0.004 | 0.000 | 0.733±0.008 | -0.004 |
|  | Black | 0.744±0.003 | 0.000 | 0.252±0.009 | 0.013 | 0.738±0.005 | 0.001 |
|  | Hispanic | 0.743±0.006 | -0.001 | 0.253±0.004 | 0.014 | 0.729±0.006 | -0.009 |
|  | Asian | 0.742±0.005 | -0.002 | 0.233±0.006 | -0.005 | 0.737±0.004 | 0.000 |
|  | Other/Uknown | 0.755±0.003 | 0.010 | 0.237±0.007 | -0.001 | 0.748±0.003 | 0.010 |
| BIO-LLAMA-3 | Male | 0.794±0.004 | 0.007 | 0.175±0.005 | 0.001 | 0.799±0.006 | 0.005 |
|  | Female | 0.787±0.005 | -0.007 | 0.174±0.006 | -0.001 | 0.794±0.005 | -0.005 |
|  | 0-25 | 0.782±0.006 | -0.007 | 0.184±0.004 | 0.005 | 0.789±0.003 | -0.005 |
|  | 26-50 | 0.799±0.005 | 0.010 | 0.175±0.003 | -0.005 | 0.797±0.007 | 0.004 |
|  | 51-75 | 0.796±0.004 | 0.007 | 0.187±0.006 | 0.007 | 0.790±0.002 | -0.004 |
|  | 75 plus | 0.782±0.003 | -0.007 | 0.169±0.003 | -0.011 | 0.797±0.008 | 0.004 |
|  | White | 0.799±0.006 | 0.013 | 0.170±0.007 | -0.001 | 0.788±0.005 | 0.007 |
|  | Black | 0.786±0.005 | 0.000 | 0.164±0.002 | -0.008 | 0.781±0.005 | 0.000 |
|  | Hispanic | 0.780±0.003 | -0.006 | 0.179±0.005 | 0.007 | 0.790±0.004 | 0.009 |
|  | Asian | 0.786±0.006 | 0.000 | 0.172±0.004 | 0.000 | 0.780±0.005 | -0.001 |
|  | Other/Uknown | 0.789±0.006 | 0.003 | 0.174±0.005 | 0.002 | 0.773±0.006 | -0.009 |

Table 3. Performance and performance disparities for models utilizing few-shot ICL approach (4 annotated data per class).

| **Model** | **Attributes** | **TPR** | **TPR**  **Disparity** | **FPR** | **FPR**  **Disparity** | **F1** | **F1**  **Disparity** |
| --- | --- | --- | --- | --- | --- | --- | --- |
| LLAMA-3 | Male | 0.824±0.004 | 0.003 | 0.193±0.003 | 0.006 | 0.812±0.005 | -0.005 |
|  | Female | 0.821±0.006 | -0.003 | 0.187±0.004 | -0.006 | 0.816±0.006 | 0.005 |
|  | 0-25 | 0.829±0.004 | -0.001 | 0.187±0.003 | 0.007 | 0.821±0.007 | -0.004 |
|  | 26-50 | 0.829±0.007 | -0.001 | 0.173±0.002 | -0.007 | 0.822±0.008 | -0.003 |
|  | 51-75 | 0.838±0.003 | 0.008 | 0.173±0.003 | -0.007 | 0.834±0.005 | 0.010 |
|  | 75 plus | 0.831±0.002 | 0.001 | 0.193±0.004 | 0.013 | 0.828±0.006 | 0.003 |
|  | White | 0.840±0.005 | 0.009 | 0.185±0.005 | -0.003 | 0.829±0.007 | 0.000 |
|  | Black | 0.832±0.005 | 0.001 | 0.195±0.003 | 0.007 | 0.828±0.004 | -0.001 |
|  | Hispanic | 0.831±0.004 | 0.000 | 0.189±0.006 | 0.001 | 0.831±0.008 | 0.002 |
|  | Asian | 0.830±0.006 | -0.001 | 0.184±0.003 | -0.004 | 0.846±0.006 | 0.017 |
|  | Other/Uknown | 0.818±0.004 | -0.013 | 0.188±0.003 | 0.000 | 0.828±0.005 | -0.001 |
| FLAN-T5 | Male | 0.769±0.003 | 0.004 | 0.247±0.004 | -0.007 | 0.740±0.004 | -0.012 |
|  | Female | 0.765±0.005 | -0.004 | 0.254±0.005 | 0.007 | 0.752±0.006 | 0.012 |
|  | 0-25 | 0.759±0.007 | 0.001 | 0.250±0.004 | 0.005 | 0.747±0.007 | -0.011 |
|  | 26-50 | 0.754±0.005 | -0.004 | 0.230±0.006 | -0.015 | 0.758±0.005 | 0.000 |
|  | 51-75 | 0.757±0.005 | -0.001 | 0.240±0.007 | -0.005 | 0.761±0.004 | 0.002 |
|  | 75 plus | 0.767±0.004 | 0.009 | 0.253±0.004 | 0.008 | 0.759±0.007 | 0.000 |
|  | White | 0.766±0.003 | -0.001 | 0.235±0.003 | 0.000 | 0.752±0.006 | -0.009 |
|  | Black | 0.773±0.002 | 0.006 | 0.241±0.006 | 0.006 | 0.774±0.007 | 0.013 |
|  | Hispanic | 0.772±0.005 | 0.005 | 0.246±0.005 | 0.011 | 0.762±0.005 | 0.001 |
|  | Asian | 0.753±0.006 | -0.014 | 0.229±0.004 | -0.006 | 0.761±0.004 | 0.000 |
|  | Other/Uknown | 0.767±0.006 | 0.000 | 0.231±0.006 | -0.004 | 0.760±0.006 | -0.001 |
| GEMMA-2 | Male | 0.800±0.005 | 0.002 | 0.201±0.004 | 0.005 | 0.799±0.003 | 0.008 |
|  | Female | 0.798±0.006 | -0.002 | 0.195±0.003 | -0.005 | 0.791±0.006 | -0.008 |
|  | 0-25 | 0.786±0.005 | -0.004 | 0.196±0.002 | 0.007 | 0.795±0.004 | 0.000 |
|  | 26-50 | 0.799±0.002 | 0.009 | 0.189±0.002 | 0.000 | 0.783±0.005 | -0.013 |
|  | 51-75 | 0.793±0.005 | 0.002 | 0.175±0.005 | -0.013 | 0.809±0.008 | 0.013 |
|  | 75 plus | 0.788±0.004 | -0.002 | 0.188±0.003 | 0.000 | 0.796±0.005 | 0.000 |
|  | White | 0.797±0.006 | 0.007 | 0.187±0.003 | 0.000 | 0.783±0.007 | -0.009 |
|  | Black | 0.802±0.007 | 0.012 | 0.187±0.006 | 0.000 | 0.795±0.003 | 0.003 |
|  | Hispanic | 0.783±0.004 | -0.007 | 0.202±0.003 | 0.015 | 0.806±0.005 | 0.014 |
|  | Asian | 0.789±0.003 | 0.000 | 0.202±0.004 | 0.015 | 0.792±0.007 | 0.000 |
|  | Other/Uknown | 0.782±0.006 | -0.008 | 0.184±0.003 | -0.003 | 0.789±0.006 | -0.004 |
| MISTRAL-0.2 | Male | 0.753±0.007 | -0.014 | 0.238±0.002 | -0.003 | 0.759±0.007 | -0.004 |
|  | Female | 0.766±0.005 | 0.014 | 0.240±0.005 | 0.003 | 0.763±0.009 | 0.004 |
|  | 0-25 | 0.738±0.004 | -0.017 | 0.247±0.006 | 0.016 | 0.743±0.005 | -0.016 |
|  | 26-50 | 0.749±0.003 | -0.006 | 0.236±0.005 | 0.005 | 0.755±0.006 | -0.003 |
|  | 51-75 | 0.762±0.004 | 0.007 | 0.217±0.004 | -0.014 | 0.765±0.007 | 0.007 |
|  | 75 plus | 0.762±0.007 | 0.006 | 0.226±0.003 | -0.005 | 0.762±0.007 | 0.003 |
|  | White | 0.759±0.006 | 0.0044 | 0.231±0.004 | -0.004 | 0.758±0.007 | 0.007 |
|  | Black | 0.755±0.007 | 0.000 | 0.241±0.006 | 0.006 | 0.752±0.005 | 0.000 |
|  | Hispanic | 0.748±0.008 | -0.007 | 0.249±0.007 | 0.014 | 0.744±0.004 | -0.007 |
|  | Asian | 0.748±0.004 | -0.007 | 0.229±0.005 | -0.006 | 0.745±0.003 | -0.006 |
|  | Other/Uknown | 0.768±0.008 | 0.013 | 0.235±0.003 | 0.000 | 0.765±0.008 | 0.013 |
| BIO-LLAMA-3 | Male | 0.807±0.005 | 0.008 | 0.173±0.005 | -0.005 | 0.816±0.005 | 0.010 |
|  | Female | 0.799±0.006 | -0.008 | 0.178±0.003 | 0.005 | 0.806±0.005 | -0.010 |
|  | 0-25 | 0.795±0.007 | -0.005 | 0.178±0.002 | 0.003 | 0.796±0.008 | -0.011 |
|  | 26-50 | 0.805±0.004 | 0.005 | 0.173±0.004 | -0.003 | 0.816±0.009 | 0.009 |
|  | 51-75 | 0.814±0.003 | 0.014 | 0.181±0.006 | 0.005 | 0.804±0.004 | -0.003 |
|  | 75 plus | 0.794±0.002 | -0.006 | 0.168±0.004 | -0.017 | 0.810±0.003 | 0.003 |
|  | White | 0.806±0.005 | 0.010 | 0.164±0.002 | -0.001 | 0.798±0.004 | 0.007 |
|  | Black | 0.793±0.004 | -0.004 | 0.164±0.003 | -0.001 | 0.792±0.005 | 0.000 |
|  | Hispanic | 0.789±0.006 | -0.007 | 0.180±0.004 | 0.015 | 0.798±0.006 | 0.007 |
|  | Asian | 0.797±0.005 | 0.000 | 0.166±0.005 | 0.000 | 0.789±0.007 | -0.002 |
|  | Other/Uknown | 0.797±0.007 | 0.001 | 0.173±0.006 | 0.007 | 0.774±0.003 | -0.018 |

Table 4. Performance and performance disparities for models utilizing few-shot ICL approach (8 annotated data per class).

| **Model** | **Attributes** | **TPR** | **TPR Disparity** | **FPR** | **FPR Disparity** | **F1** | **F1 Disparity** |
| --- | --- | --- | --- | --- | --- | --- | --- |
| LLAMA-3 | Male | 0.837±0.005 | -0.006 | 0.195±0.002 | 0.002 | 0.839±0.006 | -0.008 |
|  | Female | 0.843±0.004 | 0.006 | 0.193±0.003 | -0.002 | 0.847±0.009 | 0.008 |
|  | 0-25 | 0.850±0.008 | -0.003 | 0.198±0.002 | 0.002 | 0.843±0.005 | -0.006 |
|  | 26-50 | 0.851±0.006 | -0.002 | 0.195±0.001 | -0.002 | 0.846±0.003 | -0.003 |
|  | 51-75 | 0.861±0.003 | 0.008 | 0.192±0.004 | -0.005 | 0.858±0.008 | 0.009 |
|  | 75 plus | 0.855±0.009 | 0.002 | 0.203±0.003 | 0.007 | 0.851±0.007 | 0.002 |
|  | White | 0.859±0.006 | 0.01 | 0.211±0.005 | -0.005 | 0.847±0.004 | -0.004 |
|  | Black | 0.849±0.004 | 0 | 0.209±0.002 | 0.003 | 0.851±0.009 | 0 |
|  | Hispanic | 0.853±0.008 | 0.004 | 0.201±0.003 | 0.005 | 0.850±0.005 | -0.001 |
|  | Asian | 0.849±0.006 | 0 | 0.195±0.003 | -0.001 | 0.857±0.003 | 0.006 |
|  | Other/Unknown | 0.845±0.003 | -0.004 | 0.196±0.004 | 0 | 0.851±0.008 | 0 |
| FLAN-T5 | Male | 0.776±0.005 | 0.003 | 0.248±0.001 | -0.011 | 0.769±0.006 | -0.001 |
|  | Female | 0.773±0.004 | -0.003 | 0.258±0.002 | 0.011 | 0.771±0.009 | 0.001 |
|  | 0-25 | 0.769±0.008 | -0.005 | 0.250±0.002 | 0 | 0.751±0.005 | -0.023 |
|  | 26-50 | 0.774±0.006 | 0 | 0.223±0.001 | -0.026 | 0.775±0.003 | 0.002 |
|  | 51-75 | 0.773±0.003 | -0.001 | 0.250±0.005 | 0 | 0.786±0.008 | 0.012 |
|  | 75 plus | 0.779±0.009 | 0.005 | 0.251±0.003 | 0.011 | 0.771±0.007 | -0.002 |
|  | White | 0.773±0.006 | -0.008 | 0.234±0.003 | -0.007 | 0.767±0.004 | -0.006 |
|  | Black | 0.781±0.004 | 0 | 0.242±0.002 | 0.001 | 0.788±0.009 | 0.016 |
|  | Hispanic | 0.782±0.008 | 0.001 | 0.260±0.003 | 0.019 | 0.771±0.005 | -0.001 |
|  | Asian | 0.772±0.006 | -0.009 | 0.224±0.001 | -0.017 | 0.783±0.003 | 0.011 |
|  | Other/Unknown | 0.783±0.005 | 0.002 | 0.240±0.004 | 0 | 0.772±0.004 | 0 |
| GEMMA-2 | Male | 0.814±0.008 | -0.006 | 0.207±0.002 | 0.001 | 0.810±0.005 | -0.003 |
|  | Female | 0.820±0.006 | 0.006 | 0.206±0.003 | -0.001 | 0.813±0.004 | 0.003 |
|  | 0-25 | 0.813±0.004 | -0.002 | 0.207±0.004 | 0.001 | 0.815±0.009 | -0.001 |
|  | 26-50 | 0.819±0.009 | 0.004 | 0.205±0.003 | -0.001 | 0.805±0.006 | -0.011 |
|  | 51-75 | 0.807±0.005 | -0.007 | 0.192±0.002 | -0.014 | 0.826±0.003 | 0.01 |
|  | 75 plus | 0.816±0.003 | 0.001 | 0.209±0.001 | 0.003 | 0.818±0.008 | 0.001 |
|  | White | 0.821±0.007 | 0.008 | 0.193±0.002 | -0.013 | 0.801±0.009 | -0.015 |
|  | Black | 0.817±0.005 | 0.004 | 0.204±0.003 | -0.002 | 0.817±0.008 | 0.001 |
|  | Hispanic | 0.813±0.009 | 0 | 0.213±0.004 | 0.007 | 0.823±0.006 | 0.007 |
|  | Asian | 0.803±0.006 | -0.01 | 0.219±0.005 | 0.014 | 0.817±0.007 | 0 |
|  | Other/Unknown | 0.805±0.008 | -0.008 | 0.206±0.003 | 0 | 0.812±0.009 | -0.004 |
| MISTRAL-0.2 | Male | 0.775±0.005 | -0.004 | 0.254±0.001 | -0.004 | 0.766±0.009 | -0.008 |
|  | Female | 0.779±0.008 | 0.004 | 0.258±0.002 | 0.004 | 0.775±0.003 | 0.008 |
|  | 0-25 | 0.763±0.004 | -0.014 | 0.273±0.003 | 0.032 | 0.758±0.008 | -0.017 |
|  | 26-50 | 0.774±0.007 | -0.003 | 0.245±0.004 | 0.004 | 0.774±0.006 | -0.001 |
|  | 51-75 | 0.783±0.009 | 0.006 | 0.219±0.003 | -0.023 | 0.776±0.005 | 0.001 |
|  | 75 plus | 0.780±0.003 | 0.003 | 0.238±0.002 | -0.004 | 0.777±0.004 | 0.001 |
|  | White | 0.783±0.008 | 0.004 | 0.245±0.001 | -0.001 | 0.764±0.007 | -0.006 |
|  | Black | 0.776±0.004 | -0.003 | 0.269±0.005 | 0.023 | 0.771±0.003 | 0.002 |
|  | Hispanic | 0.779±0.006 | 0 | 0.274±0.002 | 0.028 | 0.761±0.009 | -0.009 |
|  | Asian | 0.776±0.009 | -0.003 | 0.234±0.003 | -0.012 | 0.770±0.004 | 0 |
|  | Other/Unknown | 0.782±0.003 | 0.003 | 0.246±0.001 | 0 | 0.773±0.008 | 0.003 |
| BIO-LLAMA-3 | Male | 0.829±0.009 | 0.005 | 0.183±0.002 | -0.005 | 0.835±0.004 | 0.01 |
|  | Female | 0.824±0.006 | -0.005 | 0.188±0.003 | 0.005 | 0.825±0.008 | -0.01 |
|  | 0-25 | 0.813±0.008 | -0.008 | 0.189±0.001 | 0.002 | 0.818±0.005 | -0.012 |
|  | 26-50 | 0.829±0.004 | 0.008 | 0.184±0.003 | -0.002 | 0.833±0.006 | 0.003 |
|  | 51-75 | 0.825±0.005 | 0.004 | 0.197±0.003 | 0.011 | 0.829±0.009 | -0.001 |
|  | 75 plus | 0.818±0.007 | -0.003 | 0.183±0.002 | -0.003 | 0.832±0.004 | 0.001 |
|  | White | 0.830±0.003 | 0.007 | 0.186±0.002 | 0.004 | 0.822±0.007 | 0.003 |
|  | Black | 0.823±0.008 | 0 | 0.179±0.005 | -0.004 | 0.812±0.006 | -0.007 |
|  | Hispanic | 0.815±0.004 | -0.008 | 0.194±0.003 | 0.011 | 0.825±0.005 | 0.006 |
|  | Asian | 0.817±0.009 | -0.006 | 0.179±0.002 | -0.004 | 0.819±0.003 | 0 |
|  | Other/Unknown | 0.823±0.006 | 0 | 0.183±0.003 | 0 | 0.813±0.008 | -0.006 |

Table 5. Performance and performance disparities for models utilizing few-shot ICL approach (16 annotated data per class).

| **Model** | **Attributes** | **TPR** | **TPR**  **Disparity** | **FPR** | **FPR**  **Disparity** | **F1** | **F1**  **Disparity** |
| --- | --- | --- | --- | --- | --- | --- | --- |
| LLAMA-3 | Male | 0.859±0.006 | 0.003 | 0.187±0.005 | 0.007 | 0.866±0.004 | 0.006 |
|  | Female | 0.856±0.004 | -0.003 | 0.180±0.006 | -0.007 | 0.860±0.003 | -0.006 |
|  | 0-25 | 0.863±0.003 | -0.005 | 0.184±0.007 | 0.009 | 0.875±0.005 | -0.003 |
|  | 26-50 | 0.868±0.007 | -0.001 | 0.166±0.004 | -0.009 | 0.878±0.006 | 0.001 |
|  | 51-75 | 0.875±0.005 | 0.007 | 0.163±0.003 | -0.012 | 0.892±0.003 | 0.014 |
|  | 75 plus | 0.869±0.004 | 0.001 | 0.190±0.004 | 0.015 | 0.877±0.004 | -0.001 |
|  | White | 0.884±0.006 | 0.012 | 0.173±0.004 | -0.008 | 0.884±0.005 | 0.010 |
|  | Black | 0.871±0.007 | 0.000 | 0.182±0.006 | 0.001 | 0.867±0.006 | -0.008 |
|  | Hispanic | 0.876±0.005 | 0.004 | 0.185±0.003 | 0.004 | 0.888±0.004 | 0.014 |
|  | Asian | 0.865±0.008 | -0.006 | 0.169±0.004 | -0.012 | 0.874±0.003 | 0.000 |
|  | Other/Uknown | 0.861±0.009 | -0.010 | 0.181±0.005 | 0.000 | 0.863±0.005 | -0.012 |
| FLAN-T5 | Male | 0.801±0.004 | -0.007 | 0.224±0.003 | -0.009 | 0.800±0.006 | -0.013 |
|  | Female | 0.808±0.006 | 0.007 | 0.233±0.004 | 0.009 | 0.813±0.006 | 0.013 |
|  | 0-25 | 0.792±0.005 | -0.001 | 0.241±0.005 | 0.010 | 0.789±0.006 | -0.020 |
|  | 26-50 | 0.790±0.006 | -0.003 | 0.220±0.003 | -0.011 | 0.808±0.004 | 0.000 |
|  | 51-75 | 0.794±0.005 | 0.001 | 0.221±0.002 | -0.010 | 0.809±0.006 | 0.001 |
|  | 75 plus | 0.801±0.007 | 0.008 | 0.240±0.004 | 0.010 | 0.809±0.005 | 0.000 |
|  | White | 0.798±0.010 | 0.000 | 0.222±0.003 | -0.001 | 0.792±0.003 | -0.012 |
|  | Black | 0.804±0.004 | 0.005 | 0.227±0.005 | 0.003 | 0.821±0.003 | 0.016 |
|  | Hispanic | 0.805±0.003 | 0.006 | 0.234±0.003 | 0.010 | 0.805±0.007 | 0.000 |
|  | Asian | 0.792±0.005 | -0.007 | 0.214±0.004 | -0.009 | 0.782±0.006 | -0.023 |
|  | Other/Uknown | 0.797±0.006 | -0.002 | 0.223±0.003 | 0.000 | 0.807±0.004 | 0.003 |
| GEMMA-2 | Male | 0.836±0.007 | -0.006 | 0.183±0.004 | -0.004 | 0.836±0.005 | -0.008 |
|  | Female | 0.842±0.005 | 0.006 | 0.187±0.003 | 0.004 | 0.844±0.006 | 0.008 |
|  | 0-25 | 0.821±0.006 | -0.007 | 0.191±0.005 | 0.008 | 0.830±0.005 | 0.003 |
|  | 26-50 | 0.841±0.008 | 0.012 | 0.184±0.003 | 0.000 | 0.824±0.008 | -0.003 |
|  | 51-75 | 0.824±0.004 | -0.004 | 0.170±0.005 | -0.013 | 0.838±0.004 | 0.011 |
|  | 75 plus | 0.832±0.003 | 0.004 | 0.183±0.006 | 0.000 | 0.822±0.003 | -0.006 |
|  | White | 0.832±0.006 | 0.000 | 0.174±0.003 | -0.004 | 0.829±0.006 | -0.001 |
|  | Black | 0.836±0.007 | 0.004 | 0.175±0.004 | -0.002 | 0.842±0.008 | 0.011 |
|  | Hispanic | 0.824±0.005 | -0.007 | 0.187±0.005 | 0.009 | 0.818±0.005 | -0.013 |
|  | Asian | 0.832±0.006 | 0.000 | 0.192±0.006 | 0.014 | 0.848±0.006 | 0.017 |
|  | Other/Uknown | 0.818±0.003 | -0.013 | 0.178±0.003 | 0.000 | 0.831±0.007 | 0.000 |
| MISTRAL-0.2 | Male | 0.796±0.004 | -0.007 | 0.216±0.004 | -0.004 | 0.798±0.008 | -0.015 |
|  | Female | 0.803±0.006 | 0.007 | 0.220±0.005 | 0.004 | 0.813±0.009 | 0.015 |
|  | 0-25 | 0.781±0.007 | -0.010 | 0.242±0.006 | 0.025 | 0.792±0.005 | -0.014 |
|  | 26-50 | 0.791±0.004 | 0.000 | 0.220±0.005 | 0.003 | 0.803±0.007 | -0.002 |
|  | 51-75 | 0.802±0.003 | 0.011 | 0.201±0.006 | -0.016 | 0.817±0.008 | 0.012 |
|  | 75 plus | 0.791±0.008 | 0.000 | 0.215±0.004 | -0.003 | 0.808±0.004 | 0.002 |
|  | White | 0.794±0.004 | 0.009 | 0.208±0.003 | -0.015 | 0.811±0.005 | 0.015 |
|  | Black | 0.784±0.005 | 0.000 | 0.236±0.003 | 0.013 | 0.797±0.009 | 0.000 |
|  | Hispanic | 0.783±0.006 | -0.001 | 0.235±0.006 | 0.012 | 0.792±0.006 | -0.005 |
|  | Asian | 0.778±0.004 | -0.007 | 0.213±0.007 | -0.009 | 0.792±0.004 | -0.004 |
|  | Other/Uknown | 0.791±0.003 | 0.007 | 0.223±0.003 | 0.000 | 0.815±0.008 | 0.018 |
| BIO-LLAMA-3 | Male | 0.850±0.006 | 0.006 | 0.159±0.005 | -0.014 | 0.862±0.002 | 0.005 |
|  | Female | 0.844±0.009 | -0.006 | 0.173±0.003 | 0.014 | 0.857±0.004 | -0.005 |
|  | 0-25 | 0.839±0.004 | -0.003 | 0.172±0.005 | 0.002 | 0.841±0.005 | -0.010 |
|  | 26-50 | 0.846±0.005 | 0.003 | 0.169±0.003 | -0.002 | 0.864±0.005 | 0.013 |
|  | 51-75 | 0.855±0.003 | 0.013 | 0.174±0.002 | 0.004 | 0.861±0.006 | 0.010 |
|  | 75 plus | 0.834±0.005 | -0.008 | 0.153±0.006 | -0.017 | 0.844±0.004 | -0.006 |
|  | White | 0.840±0.006 | 0.010 | 0.153±0.004 | -0.003 | 0.844±0.006 | 0.010 |
|  | Black | 0.831±0.003 | 0.001 | 0.155±0.003 | 0.000 | 0.834±0.007 | 0.000 |
|  | Hispanic | 0.824±0.004 | -0.006 | 0.165±0.005 | 0.010 | 0.832±0.005 | -0.003 |
|  | Asian | 0.829±0.004 | -0.001 | 0.153±0.002 | -0.002 | 0.839±0.004 | 0.005 |
|  | Other/Uknown | 0.830±0.005 | 0.000 | 0.165±0.004 | 0.009 | 0.824±0.003 | -0.011 |
